# Emerging applications for living crystallization-driven self-assembly

**DOI:** 10.1039/d0sc06878k

**Published:** 2021-02-12

**Authors:** Liam MacFarlane, Chuanqi Zhao, Jiandong Cai, Huibin Qiu, Ian Manners

**Affiliations:** Department of Chemistry, University of Victoria British Columbia Canada imanners@uvic.ca; School of Chemistry and Chemical Engineering, Shanghai Jiao Tong University Shanghai 200240 China hbqiu@sjtu.edu.cn

## Abstract

The use of crystallization as a tool to control the self-assembly of polymeric and molecular amphiphiles in solution is attracting growing attention for the creation of non-spherical nanoparticles and more complex, hierarchical assemblies. In particular, the seeded growth method termed living crystallization-driven self-assembly (CDSA) has been established as an ambient temperature and potentially scalable platform for the preparation of low dispersity samples of core–shell fiber-like or platelet micellar nanoparticles. Significantly, this method permits predictable control of size, and access to branched and segmented structures where functionality is spatially-defined. Living CDSA operates under kinetic control and shows many analogies with living chain-growth polymerizations of molecular organic monomers that afford well-defined covalent polymers of controlled length except that it covers a much longer length scale (*ca.* 20 nm to 10 μm). The method has been applied to a rapidly expanding range of crystallizable polymeric amphiphiles, which includes block copolymers and charge-capped homopolymers, to form assemblies with crystalline cores and solvated coronas. Living CDSA seeded growth methods have also been transposed to a wide variety of π-stacking and hydrogen-bonding molecular species that form supramolecular polymers in processes termed “living supramolecular polymerizations”. In this article we outline the main features of the living CDSA method and then survey the promising emerging applications for the resulting nanoparticles in fields such as nanomedicine, colloid stabilization, catalysis, optoelectronics, information storage, and surface functionalization.

## Abstract 2

The use of crystallization as a tool to control the self-assembly of polymeric and molecular amphiphiles in solution is attracting growing attention for the creation of non-spherical nanoparticles and more complex, hierarchical assemblies.

## Graphical abstract

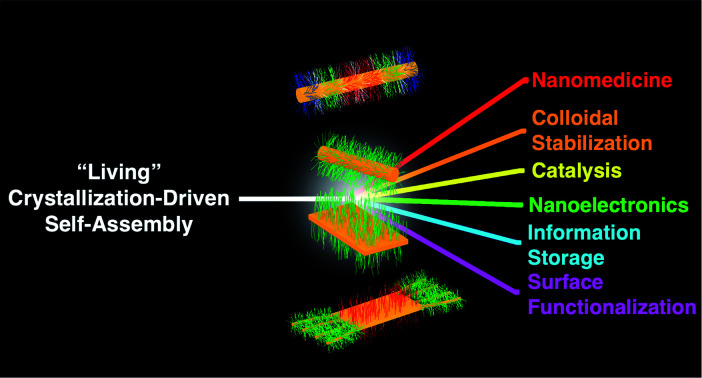

## 1. Introduction

Although molecular and macromolecular synthesis has evolved to an advanced state, the ability to prepare materials in the 10 nm to 100 micron size regime with controlled shape, dimensions, functionality, and structural hierarchy is still in its relative infancy and currently remains the near-exclusive domain of biology. Amphiphile self-assembly represents a ubiquitous approach to structures on longer length scales in Nature and is of growing importance in modern materials science. For example, it underlies the formation of cell membranes and quaternary structures from proteins as well as the widespread applications of detergents and cosmetics.^1,2^ The process is particularly well-understood in the case of low molar mass surfactants where, in solvents that are selective for the hydrophilic head group or the hydrophobic tail, different and predictable micelle morphologies can be formed based on amphiphile shape. Calculation of a ‘packing parameter’ (*P*) where 
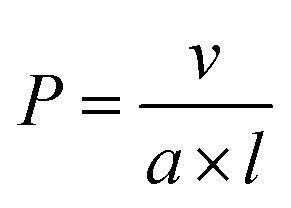
 (*a* = effective area of the solvophilic head group, *l* and *v* = length and volume of the solvophobic tail respectively) enables prediction of micelle morphology.^2^ The formation of spherical micelles is preferred for molecular surfactants with *P* ≤ 0.3, cylindrical or worm-like micelles for cases where 0.3 < *P* ≤ 0.5, and lamella (bilayers or vesicles) in the range 0.5 < *P* ≤ 1. In general, the individual surfactant molecules that comprise the resulting micelles are in a state of dynamic exchange with each other *via* low concentrations of individual, molecularly dissolved surfactant molecules, or unimers, in the surrounding solution. The existence of dynamic exchange implies that the formation of micelles from molecular surfactants represents an example of equilibrium self-assembly where the resulting nanoparticles correspond to a minimum in the free energy landscape.

**Fig. 1:**
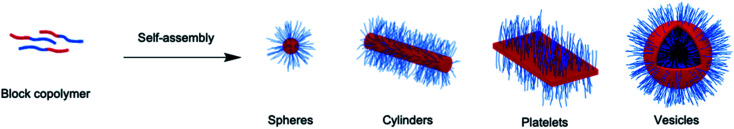
Schematic illustration of BCP self-assembly to form various core–shell nanoparticles with morphologies that are dependent on the self-assembly conditions and polymer composition.

Block copolymers (BCPs) with chemically distinct segments also display amphiphilic character and undergo self-assembly to form core–shell micellar nanoparticles when placed in a solvent that is selective for one of the blocks (see Fig. 1).^3–6^ Such micelles are relatively robust and have been very well-studied where the self-assembled core-forming block is amorphous. A classic example is polystyrene-*b*-poly(acrylic acid) (PS-*b*-PAA), which has been shown to form a range of micelles with different shapes that possess a PS core and a PAA corona in aqueous media. The observed morphologies include spheres, cylinders, vesicles, 2D lamella assemblies, and other more complex examples depending on the block ratio and specific self-assembly conditions.^7^ A similarly morphologically diverse array of self-assembled micellar nanoparticles was also accessible from the same PS-*b*-PAA BCP by modulating the coronal chain repulsions through the addition of HCl.^8^ A key general feature of the self-assembly of polymeric amphiphiles is that the micellar nanoparticles formed are kinetically trapped; exchange of block copolymer molecules between the micelles is very slow or non-existent. It is therefore much more difficult to predict the resulting nanoparticle morphologies as the packing parameter approach does not work well and they often represent non-equilibrium structures.^9^ Furthermore, as a consequence of the kinetically-trapped nature of BCP micelles, the same block copolymer can even yield different morphologies in the same solvent medium by using different processing conditions, a phenomenon that has been captured by the term “non-ergodicity”.^10,11^

Although BCP micelles are highly promising for many applications, efforts to expand their utility have suffered from several important issues. Firstly, the formation of BCP micelles with non-spherical shapes is a challenge.^9^ Many basic considerations and experimental studies (which are discussed in later sections) indicate that 1D fiber-like and 2D platelet micelles can have significant advantages over their much more common solid or hollow spherical counterparts for a wide range of applications, from uses as nanowires in devices to fields such as composite reinforcement and the delivery of therapeutic agents.^12–14^ Unfortunately, for BCPs with amorphous core-forming blocks, non-spherical micellar nanoparticles are generally only formed over a narrow range of BCP compositions and self-assembly conditions. Moreover, even when they are accessible, they are often found to be impure due to the coexistence of other morphologies. Furthermore, in cases where they are generated in a morphologically pure form, the preparation of samples of non-spherical polymer-based micellar nanoparticles with predetermined dimensions and low dispersities represents a generally unresolved challenge. This hinders their tailoring for specific applications and the achievement of uniform properties and consistent levels of performance.

A second issue is that there is limited ability to form complex micellar particles from BCPs in a predictable and controlled fashion. Impressive progress has been made in terms of generating patchy spherical nanoparticle building blocks with amorphous cores from triblock copolymers that subsequently self-assemble into a remarkable range of segmented linear chains and other extended hierarchical structures.^15,16^ However, these processes resemble step-growth polycondensation reactions and opportunities for length control and access to low dispersity samples are severely limited.

Finally, a third problem concerns the scale-up of BCP micellar nanoparticles. In most cases BCP synthesis and self-assembly are performed in separate steps, and the latter process is undertaken under conditions of high dilution. Recent work has led to major advances in this important area. By performing polymerization-induced self-assembly (PISA), where the polymerization and self-assembly steps are performed *in situ*, micellar nanoparticle concentrations of 10–50 wt% solids can be readily accessed.^17–21^ However, most of this impressive work has been accomplished with amorphous core-forming blocks where the formation of non-spherical micellar nanoparticle shapes is uncommon. Moreover, the absence of methods for predictably controlling micelle dimensions remains a key issue. For example, even in cases where the necessary time and effort has been invested to locate the optimum BCP composition and self-assembly conditions for the formation of pure cylindrical or platelet micelle phases using PISA processes, the resulting 1D or 2D assemblies generally possess unpredictable lengths or areas and high dispersities.^22^

Over the past 15 years extensive studies of the self-assembly of polymeric amphiphiles with crystallizable rather than amorphous core-forming blocks have established promising solutions to the aforementioned issues.^6,9,23,24^ These investigations have enabled the development of a wide range of 1D, 2D and complex polymer-based nanoparticles with promising scalability and potential functions that complement those available with spherical and vesicular micellar morphologies. In particular, the establishment of convenient seeded growth methods that operate at ambient temperature has allowed unprecedented control over the dimensions and architecture of micellar nanoparticles. In this perspective we describe the development and main features of this seeded growth approach, and then survey the promising emerging applications for the resulting micellar assemblies.

## 2. Crystallization-driven self-assembly

The first syntheses of well-defined BCPs were achieved *via* living anionic polymerization in the mid-to-late 1950s and studies of their solution self-assembly behavior were initiated over the following decade.^9^ Up until the early 2000s, investigations of BCP self-assembly in selective solvents were almost entirely focused on materials containing amorphous core-forming blocks. Nevertheless, several intriguing reports beginning in the mid 1960s described studies of BCPs with a crystallizable poly(ethylene oxide) core-forming block that formed 2D platelet micelles in solvents that were selective for the complementary corona-forming segment.^25,26^ By the late 1990s studies on the self-assembly of an expanded range of BCPs with crystallizable core-forming blocks had led to several further examples of 2D platelets^27–30^ and, in one case,^31,32^ 1D fiber-like micelles. Furthermore, the key role of core crystallization in the formation of micellar nanoparticles with non-spherical 1D or 2D morphologies was clearly demonstrated in 2000 through comparative solution self-assembly studies of BCPs with crystallizable poly(ferrocenyldimethylsilane)^32^ (PFDMS) segments and related BCPs with amorphous core-forming blocks.^33,34^

BCP self-assembly processes where the core crystallization plays a dominant role in the determination of the micelle morphology are now generally referred to as crystallization-driven self-assembly (CDSA). Over the past two decades, studies of the CDSA of BCPs with a crystallizable core-forming block in selective solvents have expanded dramatically. CDSA has been employed to fabricate mainly cylindrical and/or platelet micelles based on crystalline (or in some cases liquid crystalline) cores of PFDMS,^31,34–36^ poly(ferrocenylmethylsilane) (PFMS),^37^ poly(ferrocenyldiethylsilane) (PFDES),^38^ poly(ferrocenyldimethylgermane) (PFDMG),^39^ other polymetallocenes,^40,41^ poly(l-lactic acid) (PLLA),^42–48^ polycaprolactone (PCL),^49–60^ PCL/PLLA copolymers,^61^ polycarbonate,^62^ poly(ethylene oxide/glycol) (PEO/PEG),^63–65^ poly(*p*-dioxanone) (PPDO),^66–68^ polyethylene (PE),^69–83^ syndiotactic polypropylene,^84^ oligo(ethylene sulfide),^85–87^ polypeptoid,^88–93^ polyacrylonitrile,^94^ poly(2-isopropyl-2-oxazoline)^95–97^ polybis(trifluoroethoxy)phosphazene,^98,99^ poly(vinylidenefluoride),^100,101^ poly(perfluoroalkyl methacrylate),^102–105^ azobenzene-^106^ stilbene-,^107^ and cholesterol-based polymers,^108^ poly(benzylglutamate),^109^ oligopeptides,^110^ and many π-conjugated polymers such as polythiophene,^111–115^ poly(3-heptylselenophene),^116^ poly(dihexylfluorene) (PDHF),^117–119^ polyacetylene,^120,121^ oligo(*p*-phenylene),^122^ poly- and oligo(*p*-phenyleneethynylene),^123,124^ poly and oligo(*p*-phenylenevinylene).^125–128^

A typical procedure that is used for the formation of 1D and 2D micellar nanoparticles by CDSA involves heating and subsequently cooling a solution of a BCP in a solvent that is good for both blocks at elevated temperature, but which becomes selective for the corona-forming block on cooling. An alternative, well-established method involves a “solvent switch”, where dissolution of the BCP in a common solvent that is good for both blocks is followed by the slow addition of a selective solvent. In both procedures the BCP is initially completely dissolved to form unimers and, in the subsequent step, precipitation and crystallization of the core-forming block occurs, generally resulting in the formation of polydisperse fiber-like or platelet micelles.

The formation of 1D and 2D micellar nanoparticles by these two methods is postulated to involve intermediary crystalline nuclei generated during the precipitation step. It is likely that disordered/amorphous clusters containing a relatively small number of precipitated chains of the core-forming block are formed initially under conditions of poor solvation. Presumably, from these clusters, nuclei with an ordered/crystalline core emerge which are capable of initiating seeded (epitaxial) growth of further unimer (see Section 3). As the formation of crystalline nuclei by such random self-nucleation events (*i.e.* homogeneous nucleation^129^) would be expected to be slow relative to subsequent epitaxial growth, the process would be predicted to yield micelles with different dimensions that are ultimately dependent on the amount of remaining unimer and pre-existing nuclei present. For example, 1D or 2D micellar nanoparticles with reduced dimensions should be formed from new nuclei that are generated later compared to earlier in a CDSA process. The creation of a broad distribution of micellar nanoparticle lengths or areas would therefore be expected, a prediction consistent with experimental observations. Moreover, as the number of seeds formed by self-nucleation is generally relatively small, polydisperse samples containing very long 1D fiber-like micelles or large 2D platelets are typically formed under CDSA conditions.

In the overwhelming majority of cases, the use of the aforementioned CDSA methods results in 1D fibers or 2D platelets; only very rarely are spherical micelles with crystalline cores preferentially formed.^71,130^ More complex structures, such as giant vesicles, toroids, dendrites, and 3D multi-tori assemblies, have been prepared under other experimental conditions.^36,66,99,131^ The relationship between BCP structure and the resulting micellar nanoparticle morphology formed by CDSA is not entirely predictable but some general trends exist.^9^ Larger block ratios between the core- and corona-forming blocks (often *ca.* 1 : >3) usually favour the formation of 1D rather than 2D assemblies. Remarkably however, the reverse trend has also been observed in certain cases.^43,45^ Moreover, the presence of higher volume fractions of common solvent in the self-assembly medium has also been shown to favour 2D platelet formation.^132^ It therefore appears likely that generally a 2D platelet morphology is thermodynamically preferred, but that either coronal steric repulsion or low unimer solubility in the solvent medium can conspire to afford kinetically-trapped 1D (or in some cases spherical) assemblies with crystalline cores.^62^

Several studies have demonstrated that where the selective solvent chosen is very poor for the core-forming block spherical micelles with an amorphous core can be formed. In these cases it appears that precipitation of the core-forming block is so rapid that there is insufficient time for crystallization to occur.^44,132,133^ Often, over time, crystallization of the core can lead to subsequent morphological transitions to yield either fibers or platelets. More complex, non-equilibrium structures can be formed when self-assembly and core-crystallization temporally overlap.^36,66,99,131^

Combining the PISA approach with CDSA has been shown to be a promising route to scaled up synthesis of polydisperse 1D fibers and 2D platelets. This method has been demonstrated for BCPs with π-conjugated, PFDMS, PLLA, and also other organic and metallopolymer segments as crystallizable core-forming blocks.^41,107,134–139^

## 3. Living crystallization-driven self-assembly

### 3.1 The living CDSA seeded growth method

**Fig. 2:**
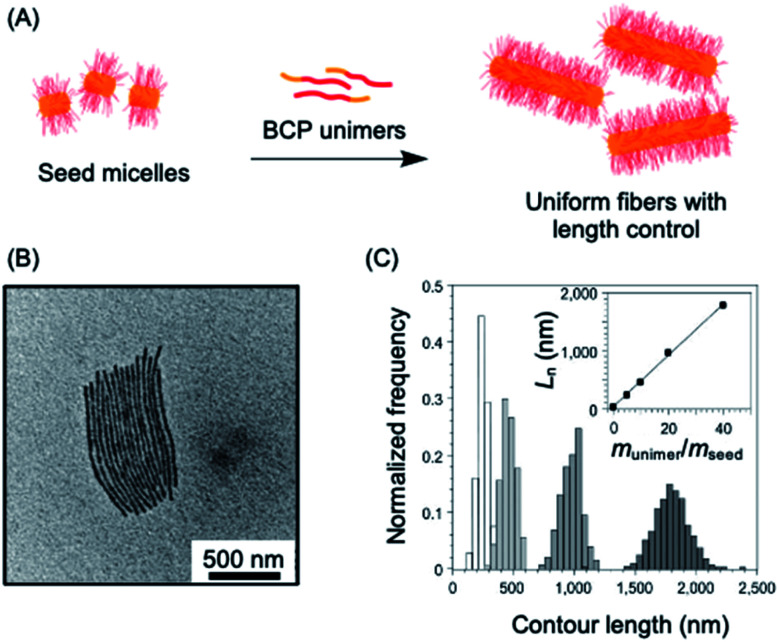
1D living CDSA. (A) Schematic illustration of the living CDSA process to generate controlled length 1D micelles from a crystallizable BCP. (B) TEM image of controlled length PFDMS-*b*-polyisoprene micelles produced by adding 20 mass equivalents of unimer to PFDMS-*b*-poly(dimethylsiloxane) seed micelles. (C) Stacked histogram plots of micelle lengths generated at different unimer to seed mass ratios, inset is a plot of average fiber length against unimer to seed mass ratios. Panels (B) and (C) reproduced from ref. 141.

A key advantage of the self-assembly of BCPs with a crystallizable core-forming block is that precise control of nanoparticle dimensions is possible using seeded-growth approaches. A consequence of the random nature of the self-nucleation process inherent to CDSA using the experimental procedures described in the previous section is that the fiber-like or platelet micelles formed possess a broad size distribution and their dimensions cannot be predicted or controlled. In 2007, it was demonstrated that, for the case of PFDMS BCPs, if the self-nucleation step is circumvented by the use of seed micelles (prepared *via* sonication-induced fragmentation of polydisperse fiber-like micelles formed *via* CDSA under self-nucleation conditions), predetermined length control was possible at ambient temperature by the addition of further unimer as the seed termini remain active to further growth (see Fig. 2a).^140^ A further development in 2010 demonstrated that when very small seeds (length *ca.* 20 nm) are used, the growth process was shown to resemble a living covalent (*e.g.* anionic) polymerization of organic monomers in that 1D micelles with excellent length control from 200 nm to 2 μm and with very low length dispersities (<1.03) could be prepared (see Fig. 2b).^141^ The increase in length detected was found to be linearly proportional to the mass ratio of added unimer to seed (see Fig. 2c). This resulted in the seeded growth method being termed “living CDSA”.

**Fig. 3:**
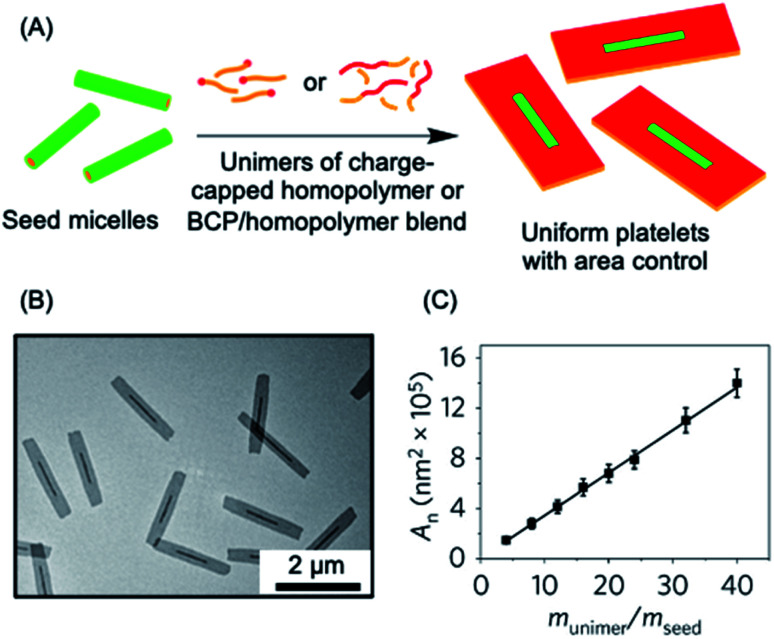
2D living CDSA. (A) Schematic illustration of the living CDSA process in 2D to generate controlled area platelet micelles by the addition of a crystallizable charge-capped homopolymer or block copolymer (BCP)/homopolymer blends to 1D seed micelles. (B) Controlled area 2D platelet micelles from the addition of 20 mass equivalents of charge-capped PFDMS homopolymer to PFDMS-*b*-poly(2-vinylpyridine) seed micelles. (C) A plot showing the linear relationship between average platelet micelle area and unimer to seed mass ratio. Panels (B) and (C) reproduced from ref. 145.

The living CDSA method has also been successfully extended to the preparation of 2D platelets, allowing ambient or near ambient temperature access to low dispersity lenticular or rectangular platelets with predictable and controlled areas based on the unimer to seed ratio.^142–144^ This has been achieved by seeded growth of either BCPs, blends of a BCP and the homopolymer corresponding to the core-forming block, or charge-terminated crystallizable homopolymers (see Fig. 3a–c).^142,144,145^

Living CDSA also provides a versatile method to create micellar nanoparticles that exhibit both complexity and hierarchical order. For example, 1D block comicelles with chemically distinct coronal segments can be prepared through the sequential addition of unimers with different corona-forming blocks to seeds.^140,146^ The resulting assemblies possess segments of precisely controllable dimensions and spatially-defined coronal chemistries and are ideal candidates for use as building blocks for further hierarchical assembly to access even longer length scales. For example, multimicron-sized supermicelles with spherical or cylindrical morphologies or 3D “superlattice” assemblies have been prepared by tailoring the lengths and arrangements of the hydrophobic and hydrophilic PFDMS BCPs with appropriate coronal functionalization.^147,148^ Non-centrosymmetric block comicelles can also be fabricated through the modification of the seed so that growth is unidirectional.^149^ “Barcode” micelles have been achieved using a PFDMS core with coronal blocks that were functionalised with different fluorescent dyes (see Section 4.5).^150^ Furthermore, segmented and gradient comicelle architectures can be generated by coassembly of two unimers with different growth rates.^151–153^ 1D triblock comicelles with segmented cores have been prepared using a heteroepitaxial growth process by the addition of BCP unimers with a crystallizable PFDMG core to seeds with crystalline PFDMS cores.^39^

In a similar manner, seeded heteroepitaxial growth of the crystallizable polymer blends in two dimensions has been successfully achieved to prepare well-defined “patchy” rectangular platelets.^154^ Multiblock platelet comicelles can be accessed through 2D living CDSA with sequential addition of different unimers.^142^ These platelets can then be converted into well-defined hollow 2D rectangular micelles of controlled size using a procedure that involves coronal cross-linking/dissolution steps.^144^

A wide variety of more complex block architectures have been fabricated through living CDSA. Scarf-like architectures were formed *via* the growth of unimers that give rise to cylindrical micelle “tassels” from tape-like platelet seeds.^39^ Hollow “scarf” micelles have been produced^155^ and multiarmed structures were prepared using homopolymer nanoparticles as seeds.^156^ Hierarchical hybrid mesostructures were obtained *via* the growth of PFDMS-*b*-P2VP (P2VP = poly(2-vinylpyridine)) cylindrical micelle arms from silica nanoparticles and carbon nanotubes.^157,158^ Branched micelles can be produced through the seeded growth of unimers with a smaller degree of polymerisation for the core-forming block.^159–161^ Other complex supermicellar assemblies can be obtained through the use of block comicelles with complementary, spatially-confined hydrogen-bonding capabilities.^162,163^ The use of multiple sequential living CDSA steps allows access to multimicron-size “windmill” micelles and related assemblies.^147,148,162–164^

The early, proof of concept work on living CDSA was performed with PFDMS as the crystallizable core-forming block. However, subsequent extensions to other crystallizable polymer systems has led to major expansion of the field and has enhanced the possibility for a wide variety of applications. Studies reported since 2011 have involved PLLA,^165–167^ PE,^168^ PFDES,^38^ and also hyperbranched poly(ether amine) capped with polyhedral oligomeric silsesquioxane,^143^ PCL,^169,170^ polycarbonate,^62,171^ and π-conjugated polymers such polythiophene,^172–175^ poly(3-heptylselenophene),^176^ PDHF,^177^ oligo(*p*-phenylenevinylene) (OPV),^178,179^ poly(cyclopentenylene-vinylene)^180^ and oligo(*p*-phenylene-ethynylene).^181^

**Fig. 4:**
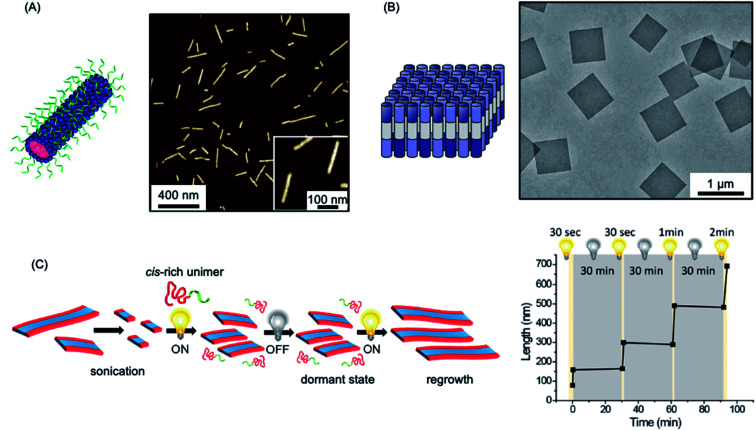
Further examples of living CDSA. (A) Schematic illustration and AFM image of 1D nanofibers comprised of amphiphilic DNA conjugates. Pink = cyanine 3 (Cy3) dyes, blue = hexaethylene chains, green = 19-mer DNA strands. (B) Schematic illustration and TEM image of 2D platelet nanostructures from the self-assembly of collagen-mimetic peptides. (C) Schematic illustration of controlled fiber formation by the living light-induced CDSA of PPV BCPs. The plot shows the increase in average fiber length over time, highlighting sample illumination times. Panel (A) reproduced from ref. 182. Panel (B) reproduced from ref. 183. Panel (C) reproduced from ref. 184.

The use of the living CDSA seeded-growth approach to fabricate well-defined biopolymer-based assemblies has also been reported. Attachment of cyanine dyes to the termini of the core-forming segments in DNA–polymer hybrids has been shown to direct self-assembly from spheres to 1D assemblies. Subsequent seeded growth experiments afforded nanofibers with controlled lengths from *ca.* 50–450 nm (see Fig. 4a).^182^ Crystallizable collagen triple helices based on collagen-mimetic peptides have been shown to undergo seeded growth to form size-tunable 2D nanosheets (see Fig. 4b).^183^ Seeded heteroepitaxial growth was also demonstrated with this system to produce segmented 2D core–shell structures.

The majority of early living CDSA experiments involved long periods of time (typically 24 h) in order to achieve complete unimer consumption. Detailed studies of the factors that influence the kinetics of living CDSA seeded growth have demonstrated that the use of longer core-forming blocks, less sterically bulky corona-forming blocks, solvents that are poorer for the core-forming block, and lower temperatures can reduce the time required to substantially less than 1 h.^151,185^ A recent report has demonstrated the formation of fibers with a crystalline π-conjugated core *via* living CDSA in under 20 min^180^

Living CDSA also appears to be very promising in terms of scalability. Combining the PISA approach with living CDSA has allowed the scalable preparation of low dispersity fiber-like micelles and block comicelles at high concentrations. These processes involved synthesis of the BCP and parallel self-assembly *in situ* in the presence of seeds.^136,139^

A fascinating extension of the living CDSA seed growth approach is to modulate the process with light. The photocontrolled living CDSA method exploits the light-induced isomerization of a poly(*p*-phenylenevinylene) (PPV) core-forming segment in a BCP (see Fig. 4c).^184^ Initially, after synthesis by ring-opening metathesis polymerization, the PPV core-forming block has a *cis* configuration of the C=C bonds in the main chain, preventing the crystallization of the core-forming block. Photoisomerization of the *cis* double bonds to the *trans* isomers by irradiation with visible light simultaneously reduces solubility and enables crystallization to occur. Length control was demonstrated in the presence of seeds by altering light irradiation times, and hierarchical structures, including block and gradient comicelles, were synthesised through the use of an additional unimer.

A reversible redox-responsive self-assembly process has been demonstrated with a polytellurophene-containing conjugated block copolymer.^186^ The selective oxidation of the polytellurophene segment, which then acted as the crystallizable core-forming block, led to 1D micelles with a narrow length distribution. The *in situ* generation of seeds *via* oxidation was proposed and the formation of 1D fiber-like micelles with a narrow length distribution was suggested to occur *via* the subsequent addition of unoxidized unimer through a living CDSA seeded growth process.

### 3.2 Variants of the living CDSA process: thermal and solvent-induced self-seeding

The seeded growth process discussed above (Section 3.1) represents the most easily controlled and reproducible living CDSA procedure. The process operates at or near ambient temperature and can be used to prepare low dispersity 1D and 2D nanoparticles from a wide variety of BCPs and charge-capped homopolymers and, as discussed below, also π-stacking molecular amphiphiles (see Section 3.3). A key feature of living CDSA is that the dimensions of the particles formed can be predetermined as they depend on the unimer to seed ratio, which can be easily controlled experimentally. However, an important variant of this process is thermal self-seeding, a method first discovered in the mid 1960s for preparing low dispersity polymer crystals.^187,188^ A more in depth understanding of the mechanism was provided by detailed studies in 2009, and also more recently.^189,190^ In contrast to the case of the living CDSA seeded growth approach, in self-seeding experiments the seeds used to induce growth of unimer are generated thermally *in situ* in solution, typically at 50–100 °C.^187–192^ When applied to 1D fiber-like micelles the approach typically involves the mild sonication of a sample of long polydisperse 1D precursor micelles, formed by self-nucleation, to form shorter micelles which are normally longer than those used as seeds in living CDSA seeded growth protocols.^191,192^ Thermal annealing of the resulting micelle fragments at a constant elevated temperature then results in selective dissolution of the less-ordered regions present in the micelle cores which effectively exhibit a lower melting temperatures. On subsequent cooling, the unimers released crystallize upon the surviving, more highly crystalline residual micelle fragments which function as seeds, thereby leading to the formation of low dispersity fiber-like micelles. In a thermal self-seeding experiment the number of surviving seeds, and therefore the unimer-to-seed ratio, can be varied through careful modulation of the solution temperature. The higher the temperature, the fewer the number of surviving seeds, and therefore the higher the effective unimer-to-seed ratio, and the longer the resulting 1D micelles subsequently formed on cooling.

The thermal self-seeding technique has been successfully used to form 2D single crystals, not only from crystallizable homopolymers, but also analogous end-functionalized homopolymers and BCPs.^193–195^ Significantly, it has been successfully applied to several systems where ambient temperature living CDSA seeded growth methods have been of limited success. For example, during living CDSA seeded growth experiments with π-conjugated BCPs with crystallizable core-forming blocks such as poly(3-hexylthiophene) (P3HT), rapid crystallization has been suggested to lead to the build-up of crystal defects, which are believed to hinder controlled fiber growth.^174,175^ Initial reports of living CDSA of P3HT-containing BCPs by seeded-growth were limited to lengths of *ca.* 250–300 nm.^175,196^ In contrast, low dispersity fibers produced by the thermal self-seeding method can exceed 1 μm in length, an improved result that has been attributed to a reduction in the number defects in the core due to the elevated temperatures used.^197,198^ Thermal self-seeding has also been applied successfully to allow the preparation of 1D fibers with a liquid crystalline perfluorinated core.^103,199^ In this case attempts to induce seeded growth by living CDSA led to competing formation of new seeds by self-nucleation, which is favoured at ambient temperatures. The thermal self-seeding method has also been productively used for the formation of 1D and 2D micelles from BCPs based on PEO,^200^ PCL^201^ and OPV,^202^ in addition to 2D platelets from PLLA^45,203^ BCPs.

Although the thermal self-seeding method is very convenient, and has important utility, compared to the living CDSA seeded growth approach, it suffers from a series of disadvantages with respect to the controlled and reproducible micellar nanoparticle preparation. These include more challenging length control as a very large dimensional variation for the resulting micelles generally occurs over a relatively small temperature range. Moreover, for BCPs with the same segment chemistries, differences in the length-temperature correlation plots exist, not only in cases of dissimilar block ratios and/or overall molar mass, but also for identical samples due to dependence of the degree of crystallinity on thermal history. Thus, for any given crystallizable polymer sample the relationship between the length of the fibers and temperature cannot be predicted in advance and needs to be established experimentally. Recent experimental observations and theoretical modeling have also indicated that, in most cases, the length *versus* temperature plot would also be expected to depend on concentration,^204,205^ a result that also potentially complicates scale up procedures for uniform micelles with a targeted length. Finally, although it is possible to access more complex polymer architectures such as block comicelles *via* thermal self-seeding protocols,^206^ this is considerably more challenging than for living CDSA seeded growth methods. In the latter case, relatively simple procedures involving sequential unimer addition to seeds can be used and the results are predictable in advance and the resulting structures exquisitely controlled.

A potentially very useful but, at present, considerably less explored variant of self-seeding involves the addition of a common solvent to micelles in a selective solvent in order to induce partial dissolution.^191^ This solvent-induced self-seeding approach offers the advantage of allowing the nanoparticle formation process to operate at ambient rather than elevated temperature, but otherwise suffers from similar disadvantages as the analogous thermal process relative to living CDSA seeded growth methods in terms of control and reproducibility.

### 3.3 Living CDSA with π-stacking molecular amphiphiles: living supramolecular polymerizations

Many molecular amphiphiles form π-stacked or hydrogen-bonded 1D (and less commonly 2D) assemblies, which are regarded as examples of supramolecular polymers due to the presence of directional, non-covalent interactions between the monomer units.^207,208^ Such systems are generally dynamic and represent excellent examples of equilibrium self-assembly under thermodynamic control.^209^ However, many amphiphilic molecular species form kinetically-trapped assemblies *via* nucleation–elongation processes that can exist over substantial periods of time under easily accessible experimental conditions.^210–212^ This opens the door to the possibility of applying the living CDSA seeded growth concept developed for crystallizable polymeric amphiphiles to appropriate molecular analogues providing that kinetic control can be maintained. The development of “living supramolecular polymerizations” of this type has allowed access to a growing range of low dispersity 1D and 2D assemblies with controlled dimensions as well as more complex architectures in kinetically-trapped states.^212–218^

**Fig. 5:**
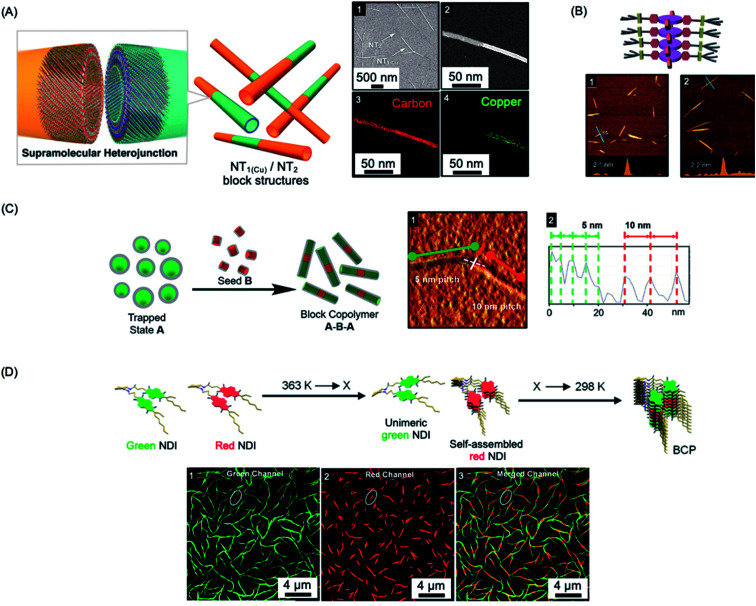
Living CDSA/living supramolecular polymerizations of amphiphilic π-stacking amphiphiles. (A) Supramolecular self-assembly of amphiphilic substituted hexabenzocoronenes to form heterojunction nanotubes (NT). Sample imaged by SEM (1) and scanning TEM (2) with energy-dispersive X-ray elemental mapping of a fiber showing carbon (3) and copper (attached to the substituents) (4) located on the nanofiber. (B) Schematic illustration and AFM micrographs of controlled length self-assembled zinc porphyrin fibers after one (1) and two (2) growth cycles. (C) Schematic illustration and AFM micrograph (1) of segmented helical self-assembled perylene bisimide fibers formed by seeded growth. Each block can be distinguished by a varying pitch distance as observed by AFM height mapping (2). (D) Supramolecular BCP structure formation through thermally-controlled stepwise aggregation of naphthalene diimide (NDI) dimers, X = elongation temperature of the red emissive NDI dimer. Block-like structure visualised by structured illumination microscopy (1–3). Panel (A) reproduced from ref. 219. Panel (B) reproduced from ref. 220. Panel (C) reproduced from ref. 221. Panel (D) reproduced from ref. 222.

The first steps towards a living supramolecular polymerization were made in 2011 and involved a system based on hexabenzocoronene-based amphiphiles. Growth from seed fibers allowed the preparation of kinetically-stable segmented structures with different peripheral substituents and band gaps that functioned as supramolecular heterojunctions (see Fig. 5a).^219^ Seeded growth approaches were studied in detail for systems based on zinc porphyrins amphiphiles in 2014 with impressive length control of the resulting low dispersity fibers evidenced for the first time (see Fig. 5b).^220,223,224^ Kinetic studies revealed 1^st^ order growth behavior with respect to the zinc porphyrin amphiphile which is analogous to that expected for a conventional living chain-growth covalent polymerization.

Other examples of molecular amphiphiles that undergo living supramolecular polymerization include amphiphilic square planar Pt(ii) phenanthroline and related complexes,^225–229^ perylene bisimides,^221,230^ and a wide range of additional π-stacking and hydrogen-bonding species.^210,231,232^ Complex architectures, such as well-defined supramolecular block copolymers, have also been successfully prepared and their study represents an exciting new area within the flourishing field of supramolecular polymers.^221,222,229,233,234^ For example, A–B–A supramolecular block copolymers of amphiphilic perylene bisimides have been fabricated by seeded living polymerization (see Fig. 5c).^221^ Cooperative supramolecular block copolymerization has been achieved based on naphthalene diimide monomers with optically distinct green and red fluorescence (see Fig. 5d).^235^ Seeded polymerization of cyclic, hydrogen-bonded monomers has also been described, providing a remarkable, first example of a living supramolecular polymerization involving a ring-opening process.^236^

Living supramolecular polymerization methods have also been used for hexabenzocoronene amphiphiles to form chiral nanotubes, including examples with segmented structures.^237^ Using chiral seeds, achiral perylene diimides can used to form nanotubes with the same helical bias,^238^ and hierarchical assemblies that involve, for example, the growth of 1D fibers from 2D seeds have also been prepared.^238–241^

A key requirement for the maintenance of low dispersities and the presence of well-defined blocks and complex architectures is the absence of significant dynamic behavior which would otherwise lead to equilibration. To date, detailed studies of the dynamics of assemblies formed by living supramolecular polymerizations have been rare, although in the case of low dispersity fibers prepared from cofacially stacked amphiphilic Pt(ii) complexes experiments revealed the presence of slow dynamic exchange that led to broadening of the length distributions of initially low dispersity fibers after 48 h.^226^

## 4. Applications of particles accessible using living CDSA methods

The low dispersity 1D and 2D micellar nanoparticles now accessible through living CDSA methods are attracting growing attention with respect to their potential uses. An expanding range of promising applications have been recently identified in areas such as nanomedicine, emulsion stabilizers, catalyst supports, optoelectronics, and surface functionalization, and these are now surveyed.

### 4.1 Nanomedicine

The ability to control the nanoparticle dimensions and shape through the living CDSA process gives a potential advantage in biomedical applications as both parameters have been convincingly shown to have an important influence on interactions with biological systems.^242–247^ For example, compared to spherical structures, relatively polydisperse 1D fiber-like micelles with different length ranges have been demonstrated to have different circulation times and uptake rates by cells.^243,248,249^

**Fig. 6:**
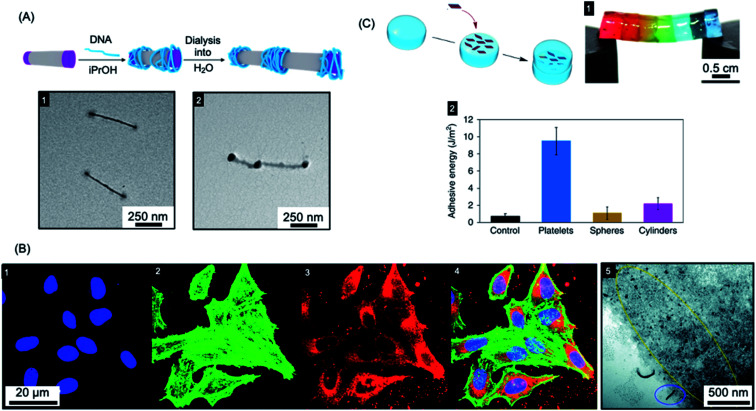
Applications of BCP nanoparticles prepared by living CDSA in nanomedicine. (A) Schematic illustration of DNA (blue) complexation and subsequent collapse at the quaternized P2VP corona (purple) of a water-soluble cylindrical micelle. TEM images of the quaternized P2VP micelles in iPrOH (1) and DNA complexed micelles after dialysis into water (2). (B) Overlaid laser scanning confocal microscopy images of cells incubated with polyfluorene fiber-like micelles functionalized with tracking (BODIPY dye) and targeting (folic acid) groups (4). Nuclei stained blue (1), F-actin stained green (2) and micelle emission red (3). TEM image of fiber-like micelle uptake (5) showing end-on interactions with the cell membrane (circled in blue) and micelle fragments (circled in yellow). (C) Platelet micelles with PLLA cores used as adhesives for thick discs of calcium-alginate hydrogels. The hydrogel discs demonstrated enhanced resistance to breaking under strain (discs coloured with different dyes) (1). The adhesive energy from bulk shear for these hydrogels far exceeded those prepared using spherical and cylindrical morphologies or adhered with water as a control (2). Panel (A) reproduced from ref. 250. Panel (B) reproduced from ref. 251. Panel (C) reproduced from ref. 252.

Colloidal stability of the micelles in water is required to allow for potential biomedical applications. This has now been achieved for a variety of crystallizable core-forming blocks, such as PFDMS,^250,253,254^ polycarbonate,^62^ PDHF,^251^ PCL,^169,255^ PLLA,^252,256–258^, PE,^70,72^ OPV.^259^ Water-soluble low dispersity triblock fiber-like comicelles with a PFDMS core have also been prepared by living CDSA.^250^ Examples with quaternized P2VP terminal segments were able to complex DNA through electrostatic interactions (see Fig. 6a). In addition, uniform 1D fiber-like micelles with a range of lengths from *ca.* 200–1800 nm have been fabricated with a PFDMS core and a corona block consisting of a statistical copolymer of (aminopropyl)-methacrylamide (APMA) and oligo(ethylene glycol methacrylate) (PFDMS_27_-*b*-PAPMA_3_-*stat*-OEGMA_48_).^253^ The APMA groups provide functionality for the attachment of drugs or metal chelators for radioimmunotherapy applications. Cell viability assays showed that the micelles are nontoxic to two human breast cancer cell lines (MDA-MB-231 and MDA-MB-436 up to a concentration of 0.1 mg mL^−1^). Fiber-like micelles with a biodegradable polycarbonate core-forming block and a PEG corona have also been prepared using living CDSA and these show no discernible cytotoxicity to either healthy (WI-38) or cancerous (HeLa) cell types.^62^

A further study using the fiber-forming PFDMS_27_-*b*-PAPMA_3_-*stat*-OEGMA_48_ materials showed that both shape and size were found to have an important influence on cellular uptake and penetration into multicellular tumor spheroids formed by the same cell lines.^260^ Low dispersity fiber-like micelles with a fluorescein label with an elongated shape and short length (80 nm) demonstrated the highest cell-uptake and the deepest penetration into the tumor models.

Segmented C–B–A–B–C pentablock 1D nanofibers were fabricated from BCPs with a PDHF core-forming segments and PEG coronal blocks that were functionalised with different terminal groups.^251^ Micelle tracking can be achieved by attaching a far-red BODIPY fluorophore to the end-group of the PEG in the B segments (see Fig. 6b). In addition, folic acid was attached to the terminal C segments to give micelles with spatially distinct functionality. A cellular uptake assay was conducted, which showed that these nanofibers were taken up through the receptor-mediated endocytosis pathway (see Fig. 6b). The micelles interacted with the cell membrane predominantly in an end-on fashion, and then subsequently localized to the perinuclear region.

Early work on polydisperse 2D sheets with a crystalline PCL cores indicated selective cell internalization when co-cultured with different cell types.^255^ In 2017, living CDSA was achieved in water for the first time with a PCL-based triblock terpolymer.^169^ By taking advantage of living CDSA in aqueous and cell culture media, a robust and biocompatible hydrogel was prepared with fibers of length >2 μm. This was successfully used to encapsulate living cells with >95% cell viability after 4 days.

Mannose-functionalized 1D cylindrical glycoparticles with biodegradable PLLA core-forming blocks have been shown to exhibit good biocompatibility.^258^ Platelet-like assemblies were examined with respect to their macrophage activation efficiency and were found to induce a more efficient inflammatory response than cylindrical analogues. Moreover, smaller glycoplatelets demonstrated higher stimulating efficiency suggesting possible future potential applications in immunology.^256^ The size of 2D platelets comprising PLLA-*b*-PDMAEMA (PDMAEMA = poly(dimethylaminoethyl methacrylate)) with coronal quaternization has also been found to affect their inhibition of bacterial growth.^257^ A detailed study found that small platelets have higher antibacterial activity compared to large platelets or spherical particles. The PLLA-*b*-PDMAEMA based 2D nanoparticles prepared by living CDSA were also shown to function as an adhesive. Furthermore, a significant enhancement of adhesion and mechanical strength was found compared to nanoparticle glues containing spherical or fiber-like micelles (see Fig. 6c).^252^ These biocompatible materials are potentially useful in a wide range of applications, including drug delivery and tissue engineering.

### 4.2 Colloid stabilization

**Fig. 7:**
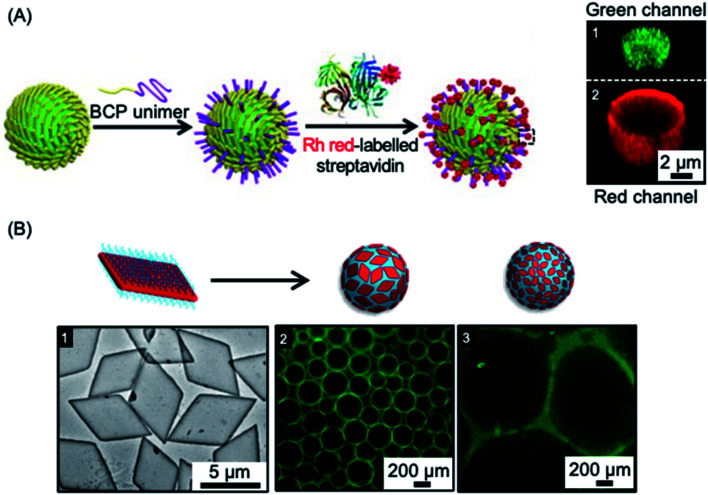
Colloidal stabilization of emulsions using 1D and 2D nanoparticles prepared *via* living CDSA methods. (A) Schematic illustration of a fiber-like micelle-stabilized oil-in-water emulsion droplet undergoing surface functionalization by growth of BCP unimers from the surface-confined fibers and subsequent functionalization with rhodamine (Rh) tagged streptavidin. Z-stacked confocal fluorescence micrographs reveal the encapsulated fluorescein isothiocyanate-dextran (green) (1) and surrounding shell of rhodamine tagged BCP (red) (2). (B) Droplet stabilization by PLLA based platelet nanostructures. TEM image of large area platelets (1), fluorescence microscopy images of emulsion droplets contain fluorescein-labelled dextran stabilised by large (2) and small (3) area platelet micelles after 60 minutes. Panel (A) reproduced from ref. 254. Panel (B) reproduced from ref. 203.

Colloidosomes are microcapsules in which the droplet interface is stabilized by colloidal particles. They have diverse applications in areas such as microencapsulation, controlled release, and catalysis, and have also been investigated as synthetic protocells with membrane-gated enzyme reactivity.^261–269^ The formation of colloidosomes was reported using fiber-like micelles of different length prepared *via* living CDSA as stabilisers of a water-in-oil Pickering emulsion.^254^ The size-specific fiber-like micelles were prepared from BCPs with appropriate wettability and chemical reactivity that consisted of a crystallizable PFDMS core and a carboxylated poly(methylvinylsiloxane) corona. After obtaining short cylindrical micelles, water-in-oil Pickering emulsions were prepared through mixing water or aqueous solution together with the assembled fiber-like micelles. The Pickering emulsion droplets ranged from 1.8 to 10.8 μm in average diameter, subsequent crosslinking of the assembled colloidosomes was achieved *via* the amidation of the coronal carboxyl groups. Furthermore, as a result of the activity of the termini of PFDMS cores of the fiber-like micelles to epitaxial growth, the cross-linked colloidosome membranes could be further functionalized to give arrays of biotinylated side chains capable of streptavidin binding (see Fig. 7a).

Another study showed that the interfacial activity of patchy worm-like micelles prepared from (PS-*b*-PE-*b*-PMMA) (PMMA = poly(methyl methacrylate)) triblock terpolymers through CDSA was comparable to that of Janus cylinders with similar dimensions and corona composition.^270^ These study suggests promising potential applications of these and related robust colloidosomes in areas of biomimetic encapsulation, drug delivery, catalysis, and biosensing.

In other work, 2D platelets prepared *via* living CDSA methods have been employed as stabilizers of water-in-water emulsions (see Fig. 7b). The effect of platelet size on emulsion stabilization was studied for materials with a crystalline PLLA core and a poly(aminomethacrylate) corona.^203^ The results demonstrated that larger size platelets were more effective at stabilizing emulsions than smaller platelets with lower surface area. It was suggested that larger size and higher surface area gives increased adsorption as well as a larger rotational barrier to the platelet particles. The work demonstrates that the ability to prepare 2D platelets of different size provides a potential route to the optimization of interfacial stabilizers for range of applications.

### 4.3 Catalysis

**Fig. 8:**
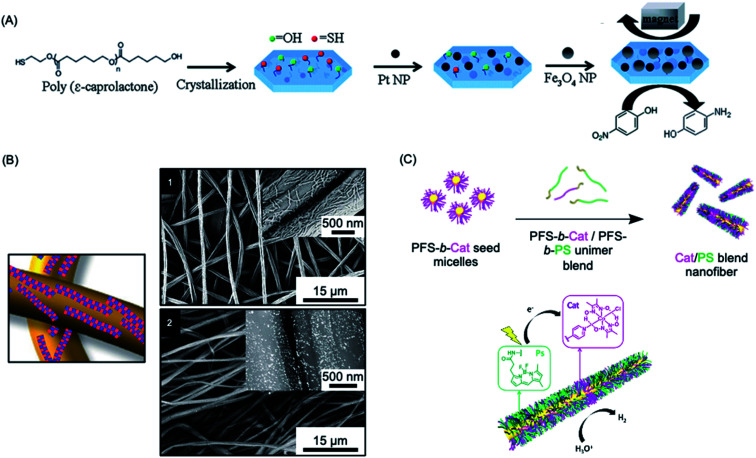
Catalysis using nanoparticles (NPs) prepared *via* living CDSA methods. (A) Schematic illustration of PCL crystallization and binding of platinum and iron oxide NPs to generate catalytically active and magnetically recoverable nanostructures. (B) Coaxially electrospun polystyrene and patchy micelles formed by CDSA which contain gold nanoparticles. SEM images (1) showing micelle coverage can be analysed with a back scattered electron detector which shows the gold NP as bright dots (2). (C) Schematic illustration of living CDSA of PFDMS BCPs with catalyst and photosensitizer moieties attached to the corona-forming block to give catalytically active nanofibers capable of hydrogen generation from water in the presence of visible light. Panel (A) reproduced from ref. 271. Panel (B) reproduced from ref. 272. Panel (C) reproduced from ref. 273.

Heterogeneous catalysis with supported nanoparticles is of major interest from the standpoint of facile product separation but loss of catalytic activity is a significant problem. In 2012, a magnetic recyclable support was described consisting of 2D homopolymer platelets derived from PCL with –OH and –SH end groups.^271^ The platelets were fabricated using a thermal self-seeding approach and the functional groups were presented on the platelet surface (see Fig. 8a). These groups can be utilized to attach to both catalytic platinum nanocatalysts and magnetically responsive Fe_3_O_4_ nanoparticles. The reduction of 4-nitrophenol to 4-aminophenol by sodium borohydride mediated by this heterogeneous catalyst illustrated the efficient catalytic activity arising from the high specific surface area of the platelets.

More recently, an efficient and reusable 1D heterogeneous catalyst system was fabricated by the coaxial electrospinning of polystyrene (PS) and patchy nanofibers prepared *via* CDSA which were subsequently loaded with Au nanoparticles (NPs) (see Fig. 8b).^272^ The patchy nanofibers attached to the PS core were fabricated by CDSA using a triblock terpolymer with a crystallizable PE central block and terminal blocks consisting of PS and amino-functionalized poly(methyl methacrylate) which phase separate in the corona.^76,274^ The functional corona patches of the nanofibers contained diisopropylamino groups which were selected to bind and efficiently stabilize the added Au NPs. The catalytic alcoholysis of dimethylphenylsilane by the electrospun AuNP-loaded nonwoven system was comparable to Au nanoparticles encapsulated in 700 nm diameter polymer microtubes prepared by a chemical vapour deposition approach, even with a lower catalyst-loading.^275^ The absence of gold leaching or reduction in reaction efficiency, even after ten cycles, demonstrated the excellent reusability and robustness of this catalytic platform. Further studies showed that even higher reactivity could be achieved by tuning the patch shapes and sizes.^276^

Photocatalytic hydrogen production offers a very promising approach to the conversion of solar energy into a chemical fuel. In 2020, a high-performance photocatalytic core–shell nanofiber system fabricated through living CDSA was reported.^273^ This system combined a photosensitizer and Co catalyst held in close proximity in the blended corona of a fiber-like micelle to split water for hydrogen production using visible-light (see Fig. 8c). The catalytic nanofiber demonstrated high efficiency and reusability with turnover numbers (>7000 over 5 h) and frequency (>1400 h^−1^) and an overall quantum yield for solar energy conversion to fuel of 4.0% under optimised conditions. The system has a much higher activity than analogous homogenous solutions of the photosensitizer and Co catalyst (turnover number *ca.* 70 over 5 h), presumably due to their enforced closer proximity in the photocatalytic nanofiber corona.

### 4.4 Optoelectronics

Due to their solution processability compared to traditional semiconductors and metals, π-conjugated semiconducting polymers have been widely explored with respect to uses in photovoltaics, light-emitting devices, field-effect transistors and sensors. However, the fabrication of uniform electroactive nanofibers and platelets with high colloidal stability, predetermined size, and spatially controlled functionality has been a major challenge.^277^ The living CDSA method offers a promising approach to solve this problem. Furthermore, as discussed below, the epitaxial growth mechanism that underlies living CDSA processes leads to major potential advantages for optoelectronic performance.

**Fig. 9:**
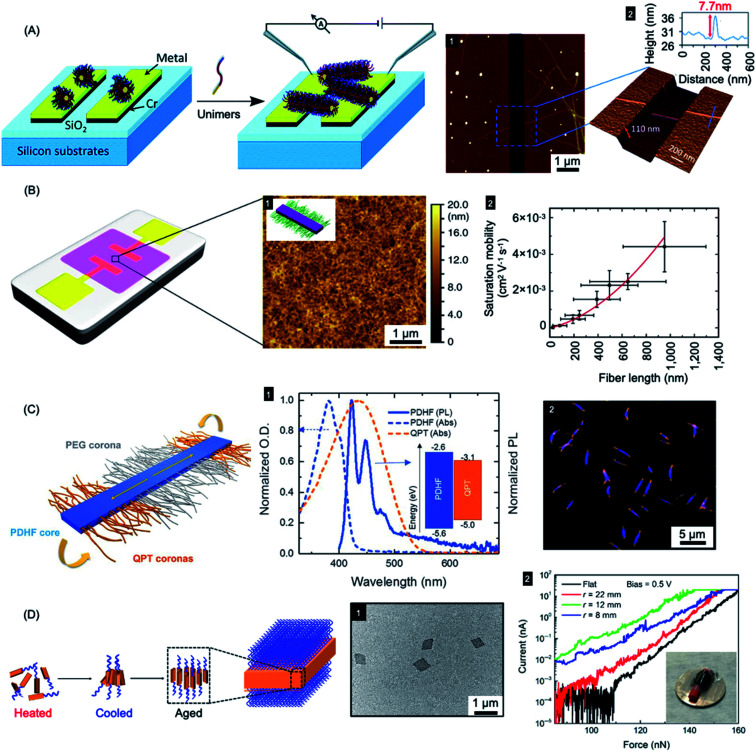
Nanoparticles prepared by living CDSA for applications in optoelectronics. (A) Schematic illustration of PFDMS-based fiber-like micelle growth across an electrode gap by living CDSA from chemisorbed seed micelles. AFM height micrograph (1) highlighting a 7.7 nm high (2) nanofiber spanning two gold electrodes. (B) Device schematic and AFM micrograph (1) of an organic field effect transistor utilizing P3HT BCP nanofibers as the active layer. A plot of saturation mobility against fiber length (2) shows a superlinear relationship. (C) B–A–B fiber-like micelles with PDHF core and a PEG and quaternized polythiophene (QPT) segmented corona. Normalised absorption and emission for PDHF and QPT (1) showing energy overlap between the PDHF emission and QPT absorption which allows for energy transfer. Laser scanning confocal microscopy image of the B–A–B micelles (2), blue = PDHF emission, orange = QPT emission. (D) Schematic illustration of PPV-*b*-P2VP self-assembly to produce 2D platelet micelles as imaged by TEM (1). Current-force characteristics (2) of a flexible pressure sensing tunnelling device (inset picture) containing a PPV-based platelet micelles with different bending radii. Panel (A) reproduced from ref. 279. Panel (B) reproduced from ref. 198. Panel (C) reproduced from ref. 177. Panel (D) reproduced from ref. 286.

Fiber-like PFDMS-*b*-P2VP micelles have been used as nanoscale templates to direct the formation of electrically conductive polyaniline.^278^ The oxidative polymerization of aniline led to the formation of a sheath of conductive polyaniline in the P2VP corona. This allowed the generation of conductive polyaniline nanofibers with precisely controlled lengths matching that of the low dispersity templates prepared by living CDSA. In a further report, a simple electrical circuit was constructed through the epitaxial growth of fiber-like micelles across an interelectrode gap (see Fig. 9a). The micelles comprised of a PFDMS core and poly(dimethylsiloxane) (PDMS) corona that was terminated with an electroactive π-conjugated poly(3-octylthiophene) segment. Subsequent oxidative doping of a thiophene block led to higher electrical conductivity.^279^

Fiber-like micelle nanowires based on a crystalline conjugated polythiophene core could be prepared with controlled lengths *via* living CDSA thermal self-seeding methods.^198^ The electroactive fibers showed excellent colloidal stability and were subsequently incorporated as an ensemble active layer in field-effect transistors (see Fig. 9b). It was demonstrated that both the degree of polymerization of the core-forming block and the fiber length had significant impact on device performance. Charge carrier mobilities were increased by fiber alignment using a surface patterning and dip-coating procedure. Conductivity of individual fibers was also demonstrated using tunnelling AFM methods.

A key problem in the use of conjugated organic polymers for optoelectronic devices such as solar cells is that excitons formed by photoabsorption are only able to diffuse 5–10 nm, a distance that is much less than the thickness needed to efficiently harvest visible light.^280^ This has a major effect on device design and performance where the lengthscales of regions of donor and acceptor materials need to be within 5–10 nm in order for excitons to reach the charge-separating interface. It is therefore of high significance that long-range exciton transport (*ca.* 200 nm) has been demonstrated in solution for individual conjugated polymer nanofibers that were fabricated *via* living CDSA.^177^ Fiber-like B–A–B triblock comicelles were prepared with a crystalline PDHF core as an energy donor, non-emissive PEG “A” segment corona, and emissive quaternized polythiophene (QPT) “B” segment coronas as energy acceptors (see Fig. 9c). These block comicelles allow for the measurement of the exciton diffusion length through the crystalline PDHF core to the lower-energy emissive QPT end block. The excitons generated along the long axes of the PDHF cores of the fiber-like micelles travel in the direction of the interchain π–π stacking. Time-resolved photoluminescence measurements showed an increase in emission intensity for the QPT (530–630 nm) which was accompanied by a reduction of the PDHF emission (∼480 nm). By changing the length of the central “A” segment it was possible to measure exciton diffusions lengths as long as *ca.* 200 nm with a large diffusion coefficient of 0.5 cm^2^ s^−1^. The long diffusion lengths were attributed to the uniform energy landscape of the highly crystalline PDHF core formed by epitaxial growth from seeds. The values suggest key potential advantages for device applications. For example, a thin film *ca.* 200 nm thick should efficiently absorb >98% of incident photons enabling the construction of efficient planar conjugated polymer-based light-harvesting devices.

Exciton diffusion has also been studied for films of fiber-like micelles with a visible light-absorbing P3HT core which were prepared with lengths up to *ca.* 2.8 μm *via* living CDSA of a phosphonium-terminated amphiphilic P3HT.^281^ Detailed photophysical studies showed that the nanofibers possess estimated diffusions lengths of 300 ± 50 nm, the highest value reported for a conjugated polymer material, and a diffusion constant of 1.1 ± 0.1 cm^2^ s^−1^.^282^

Coaxial heterojunction nanowires with controlled dimensions have been fabricated through the seeded growth living CDSA with a crystalline energy-accepting outer core-forming poly(3-(2′-ethylhexyl)thiophene) (P3EHT) block and a crystalline energy-donating PDHF inner core-forming block.^283^ It was found that Förster resonance energy transfer (FRET) from the PDHF inner core to the lower energy P3EHT outer core was enhanced compared to the analogous coaxial structure in which the P3EHT block was solvated.

In addition to the promising work with 1D nanofibers with crystalline π-conjugated cores, 2D platelet assemblies have been prepared and studied based on conjugated fluorene-containing homopolymers and P3HT-*b*-PEG diBCPs.^284,285^ Different shapes, such as rectangular, raft, and leaf-like structures could be obtained in different solvents. Uniform 2D rhombic platelet micelles with controllable sizes and thicknesses using BCPs have also been reported with a crystallizable PPV core-forming blocks.^286^ These materials were explored for use in pressure sensing devices on both rigid and flexible substrates based on vertical tunnelling (see Fig. 9d). The 2D rhombic micelles showed an on-off current ratio of more than 10^4^ and a high on-state current density (6000 A cm^−2^) with high sensitivity.

Recently, single-handed helical nanofibers with controlled length and handedness have been fabricated through the living CDSA of amphiphilic BCPs composed of a crystallizable P3HT core-forming block and a corona-forming segment comprising optically active helical poly(phenyl isocyanide) segments.^172^ It was found that during the self-assembly process the chirality of helical poly(phenyl isocyanide) was transferred to the nanofibers. These structures exhibited interesting white-light emission and circularly polarized luminescence with tunable handedness and an enhanced dissymmetric factor.

### 4.5 Information storage: “barcode” micelles

**Fig. 10:**
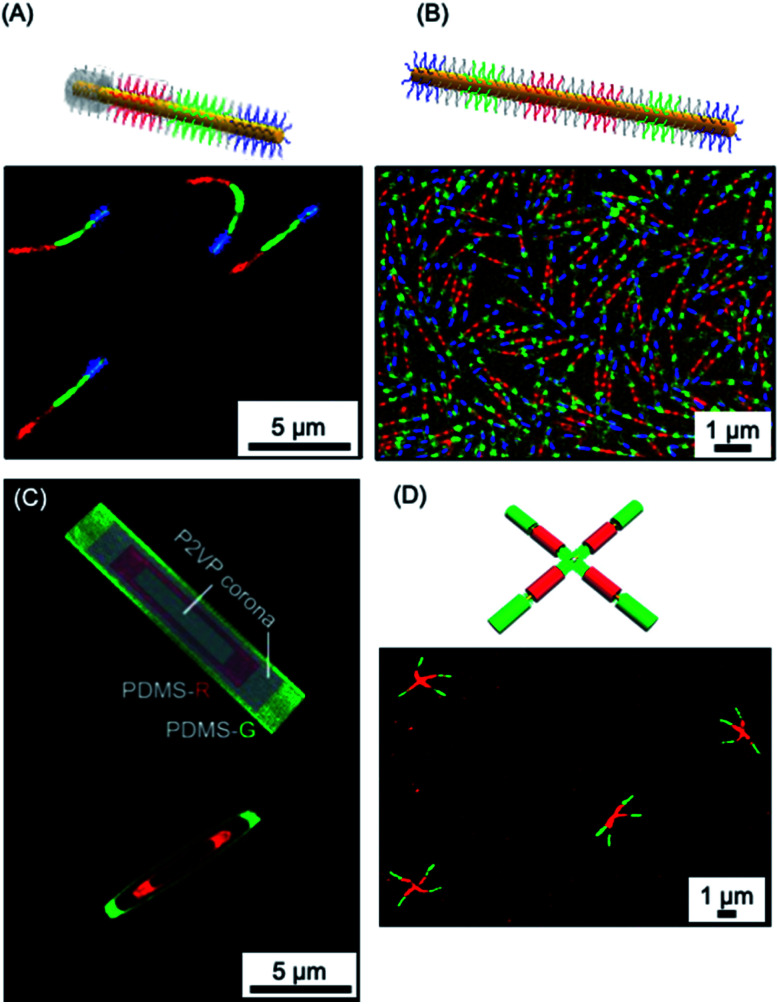
1D and 2D micellar nanoparticles with potential for information storage. (A) Laser scanning confocal microscopy image of red-green-blue fluorescent ‘nanopixels’ grown from a unidirectional cross-linked seed micelle. (B) Structured illumination microscopy image of symmetrical red-green-blue fluorescent micelles which are stable to unimer exchange after 1 year in EtOAc at ambient conditions. (C) Schematic illustration and structured illumination microscopy image of PFDMS-based platelet micelles with concentric rings of nonfluorescent P2VP and red or green dye functionalised PDMS corona chains. (D) Laser scanning confocal microscopy image of ‘cross’ supermicelles formed by hierarchically assembled segmented cylindrical micelles functionalised with red and green fluorescent dyes. Panel (A) reproduced from ref. 150. Panel (B) reproduced from ref. 150 and 288. Panel (C) reproduced from ref. 144. Panel (D) reproduced from ref. 164.

The use of living CDSA seeded growth processes that involve the sequential addition of chemically distinct unimers represents a powerful method for the convenient and efficient fabrication of segmented 1D and 2D assemblies with spatially distinct chemistries. For example, a variety of fluorescent tags have been encoded into segmented fiber-like block comicelles by sequential addition of fluorescent-labelled and non-fluorescent PFDMS-containing BCPs to the preformed micelle seeds. This yielded nanoscale barcode structures with spatially defined fluorescent colours separated by the non-fluorescent spacers (see Fig. 10a and b).^150,287^ The spatial arrangement and tuneable colours endow fluorescent barcodes with the potential for high data storage density. For example, the preparation of fiber-like block comicelles with 18 readily identifiable colours with up to seven blocks was demonstrated. By applying this platform, in principle, fiber-like micelles with 18^7^ (6.1 × 10^8^) combinations of fluorescent colour could be fabricated. Furthermore, the encoding of nanostructures with more complex patterns can be readily realized such as fluorescent non-centrosymmetric segmented comicelles^150^ (see Fig. 10a), concentric 2D block comicelles^142,144,145^ (see Fig. 10c) and hierarchical assembles^148,164^ (see Fig. 10d). 1D fiber-like micelles have been shown to be stable in solution at room temperature for over year without significant unimer exchange based on the preservation of the distinct fluorescent segments of different colour (Fig. 10b). This suggests possible applications in information storage or as calibration tools for fluorescence microscopy.^288^

### 4.6 Multifunctional micelle brushes *via* living CDSA from surfaces

**Fig. 11:**
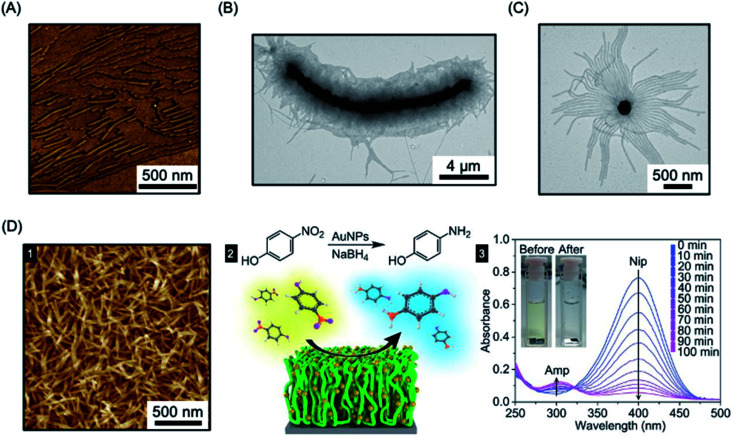
Fiber-like micelle functionalised surfaces. (A) AFM image of PFDMS-based BCP micelles growing from steps in a crystallized PFDMS homopolymer film. (B) TEM image of fibers grown from a multi-walled carbon nanotube covered in PFDMS homopolymer crystals. (C) TEM image of fibers grown from seed micelles attached to an SiO_2_ nanoparticle. (D) PFS BCP fibers grown from seed micelles immobilized on a silicon surface, imaged by AFM (1). A heterogeneous catalysis system, capable of the reduction of 4-nitrophenol (Nip) at room temperature, was generated by the attachment of AuNPs (2). The reduction to generate 4-aminophenol (Amp) could be followed by UV-Vis spectroscopy. Panel (A) reproduced from ref. 39. Panel (B) reproduced from ref. 157. Panel (C) reproduced from ref. 158. Panel (D) reproduced from ref. 289.

The “living” characteristic of CDSA allows the fabrication of controlled hierarchical assemblies through the epitaxial growth of micelles from suitably functionalised surfaces. Early work on living CDSA showed that a crystalline PFDMS homopolymer film could nucleate the growth of cylindrical micelles from the boundary of the stacked layers of crystalline lamellae to give brush structures on the surface (Fig. 11a).^39^ Following a similar rationale, multi-walled carbon nanotubes cast with PFDMS homopolymer were used to prepare a hybrid architectures with fiber-like micelles of controlled length emanating from the central scaffold (see Fig. 11b).^157^

In a different approach to micelle brushes, immobilization of crystalline BCP micelle seeds and their subsequent epitaxial growth has been shown to provide a flexible pathway to form hybrid assemblies on material surfaces. While the casting of PFDMS homopolymer appears to enable growth from π-electron-rich materials, the use of a functional corona enables effective anchoring of BCP micelle seeds onto a broad array of material surfaces. For example, PFDMS-*b*-P2VP micelle seeds have been shown to be capable of adsorbing to the surface of silica nanoparticles *via* hydrogen-bonding between the pyridyl groups of the P2VP corona and the hydroxyl groups of silica (see Fig. 11c).^158^ Further elongation was demonstrated from the PFDMS-*b*-P2VP seeds through seeded growth yielding sunflower-like assembles with excellent dispersibility and controlled size.

In a key further development, the seed attachment approach was generalised to a variety of macroscopic and planar materials surfaces. For example, attachment of seed micelles to a silicon substrate *via* spin-coating from solution provided an powerful route to create micellar brushes (see Fig. 11d) including segmented structures with tunable wettability *via* sequential unimer addition.^289^ Standing micelle arrays with a large scale areal coverage were constructed through a bottom-up living CDSA seeded growth process involving the addition of crystallizable PFDMS-*b*-P2VP unimers to a solution containing a seed-coated silicon wafer. The 3D assembly of micellar brushes was retained after drying. The density, length, and coronal chemistry of these micellar brushes could be precisely tuned, and applications in catalysis, filtration, and antibacterial surface modification were demonstrated by using post-assembly decoration with selected functional nanoparticles. Analogous to the surface-initiated covalent polymer brushes,^290^ but on a larger length scale, the creation of micellar brushes represent as a promising and versatile strategy for surface functionalization. The approach is promising not only for micellar brush growth from semiconductors such as silicon, but also metals (*e.g.* gold) and other materials (*e.g.* graphene oxide).

## 5. Outlook

Since 2000, studies of the influence of crystallization on the solution self-assembly of polymeric and molecular amphiphiles has led to the development of a rich and rapidly expanding field involving the generation of new, well-defined soft matter-based nanoparticles with emerging potential applications. Morphologies that are difficult to access, such as 1D fiber-like micelles and 2D platelets, are now readily accessible and ambient temperature seeded growth living CDSA methods yield uniform samples of nanoparticles with preselected dimensions. In addition, sequential processes involving several living CDSA steps have been used to create complex and hierarchical architectures such block comicelles and branched and scarf-like assemblies, hollow structures, and “windmill” type architectures. Seeds used for living CDSA processes can be attached to particles or surfaces to allow the grafting of 1D fiber-like micelles. Fundamental understanding of the living CDSA process can be enriched by detailed experimental studies as well as by theory,^28,204,291–294^ and builds on the foundations provided by the fields of polymer crystallization,^295^ amphiphile self-assembly,^1,2^ and supramolecular polymers.^152,208,212–218^

Future challenges include the further development of heteroepitaxial growth processes, in which crystalline core-forming blocks with different chemistries but similar lattice parameters will grow from a seed. To date, successful examples are limited,^39,154,183,219^ but further generalization will offer opportunities to access structures with of desirable combinations of properties, including heterojunctions with applications in optoelectronics. The ability of uniform 1D and 2D micellar nanoparticles to form ordered liquid crystalline phases in solution and to respond to stimuli are also areas worthy of further exploration.^296,297^ Scale up, which appears promising based on initial results, and is critical for real-world applications, is another area that requires increased future attention.

Although fascinating combinations of dissociation and fragmentation occur on heating,^189^ crystalline core-forming blocks generally yield assemblies which are “static” with respect to unimer exchange, at least in corona-selective solvents at ambient temperature. Dynamic behavior has been evidenced under ambient conditions in the cases of 1D assemblies derived from living CDSA of molecular amphiphiles^226^ and may also be accomplished more generally in cases where the core is liquid crystalline. Interesting phenomena such as linear aggregation and fusion of 1D micelles has already been noted in the case of a LC perfluorinated or poly(benzylglutamate) core.^103,109^ The more general development of systems where dynamic behavior can be “turned-on” under programmed conditions may offer new opportunities in terms of properties and also applications, for example in uses as therapeutic delivery vehicles.

As discussed in this article, recent work has unveiled potential utility for particles formed by living CDSA in a variety of areas. The ability to intricately tailor particles should also lead to important advantages in areas such as targeted therapeutics, imaging, and multistep catalysis. The growth of micelle brushes from surfaces offers new opportunities for capturing and funneling energy, preparing membranes for filtration, as well as for catalysis under flow. With the growing appreciation that shape, dimensions, as well as spatially-defined functionality can play a key role in a wide range of areas, the future of the field appears exceptionally bright.

## Conflicts of interest

There are no conflicts of interest to declare.

## References

[cit1] Wang C., Wang Z., Zhang X. (2012). Acc. Chem. Res..

[cit2] Antonietti M., Förster S. (2003). Adv. Mater..

[cit3] Förster S., Antonietti M. (1998). Adv. Mater..

[cit4] Mai Y., Eisenberg A. (2012). Chem. Soc. Rev..

[cit5] Schacher F. H., Rupar P. A., Manners I. (2012). Angew. Chem., Int. Ed..

[cit6] Deng Z., Liu S. (2020). Polymer.

[cit7] Cameron N. S., Corbierre M. K., Eisenberg A. (1999). Can. J. Chem..

[cit8] Zhang L., Yu K., Eisenberg A. (1996). Science.

[cit9] Tritschler U., Pearce S., Gwyther J., Whittell G. R., Manners I. (2017). Macromolecules.

[cit10] Hayward R. C., Pochan D. J. (2010). Macromolecules.

[cit11] Jain S., Bates F. S. (2004). Macromolecules.

[cit12] Foster J. C., Varlas S., Couturaud B., Coe Z., O'Reilly R. K. (2019). J. Am. Chem. Soc..

[cit13] Truong N. P., Quinn J. F., Whittaker M. R., Davis T. P. (2016). Polym. Chem..

[cit14] Boott C. E., Nazemi A., Manners I. (2015). Angew. Chem., Int. Ed..

[cit15] Gröschel A. H., Walther A., Löbling T. I., Schacher F. H., Schmalz H., Müller A. H. E. (2013). Nature.

[cit16] Pochan D. J., Chen Z., Cui H., Hales K., Qi K., Wooley K. L. (2004). Science.

[cit17] Derry M. J., Fielding L. A., Armes S. P. (2016). Prog. Polym. Sci..

[cit18] Wan W. M., Pan C. Y. (2007). Macromolecules.

[cit19] Penfold N. J. W., Yeow J., Boyer C., Armes S. P. (2019). ACS Macro Lett..

[cit20] Canning S. L., Smith G. N., Armes S. P. (2016). Macromolecules.

[cit21] Groison E., Brusseau S., D'Agosto F., Magnet S., Inoubli R., Couvreur L., Charleux B. (2012). ACS Macro Lett..

[cit22] Nahi O., Cayre O. J., Kim Y. Y., Smith A. J., Warren N. J., Meldrum F. C. (2020). Chem. Commun..

[cit23] Ganda S., Stenzel M. H. (2020). Prog. Polym. Sci..

[cit24] He W. N., Xu J. T. (2012). Prog. Polym. Sci..

[cit25] Lotz B., Kovacs A. J., Bassett G. A., Keller A. (1966). Kolloid Z. Z. Polym..

[cit26] Kovacs A. J., Manson J. A. (1966). Kolloid Z. Z. Polym..

[cit27] Richter D., Schneiders D., Monkenbusch M., Willner L., Fetters L. J., Huang J. S., Lin M., Mortensen K., Farago B. (1997). Macromolecules.

[cit28] Vilgis T., Halperin A. (1991). Macromolecules.

[cit29] Ramzi A., Prager M., Richter D., Efstratiadis V., Hadjichristidis N., Young R. N., Allgaier J. B. (1997). Macromolecules.

[cit30] Lin E. K., Gast A. P. (1996). Macromolecules.

[cit31] Massey J., Nicole Power K., Manners I., Winnik M. A. (1998). J. Am. Chem. Soc..

[cit32] Hailes R. L. N., Oliver A. M., Gwyther J., Whittell G. R., Manners I. (2016). Chem. Soc. Rev..

[cit33] Massey J. A., Temple K., Cao L., Rharbi Y., Raez J., Winnik M. A., Manners I. (2000). J. Am. Chem. Soc..

[cit34] Cao L., Manners I., Winnik M. A. (2002). Macromolecules.

[cit35] Wurm F., Hilf S., Frey H. (2009). Chem.–Eur. J..

[cit36] Guerin G., Cruz M., Yu Q. (2020). Sci. Adv..

[cit37] Du V. A., Qiu H., Winnik M. A., Whittell G. R., Manners I. (2016). Macromol. Chem. Phys..

[cit38] Gädt T., Schacher F. H., McGrath N., Winnik M. A., Manners I. (2011). Macromolecules.

[cit39] Gädt T., Ieong N. S., Cambridge G., Winnik M. A., Manners I. (2009). Nat. Mater..

[cit40] Sha Y., Zhang Y., Zhu T., Tan S., Cha Y., Craig S. L., Tang C. (2018). Macromolecules.

[cit41] Sha Y., Rahman M. A., Zhu T., Cha Y., McAlister C. W., Tang C. (2019). Chem. Sci..

[cit42] Fu J., Luan B., Yu X., Cong Y., Li J., Pan C., Han Y., Yang Y., Li B. (2004). Macromolecules.

[cit43] Yu W., Inam M., Jones J. R., Dove A. P., O'Reilly R. K. (2017). Polym. Chem..

[cit44] Petzetakis N., Walker D., Dove A. P., O'Reilly R. K. (2012). Soft Matter.

[cit45] Inam M., Cambridge G., Pitto-Barry A., Laker Z. P. L., Wilson N. R., Mathers R. T., Dove A. P., O'Reilly R. K. (2017). Chem. Sci..

[cit46] Song Y., Chen Y., Su L., Li R., Letteri R. A., Wooley K. L. (2017). Polymer.

[cit47] Wang Z., Cao Y., Song J., Xie Z., Wang Y. (2016). Langmuir.

[cit48] Li C., Liu R., Xue Q., Huang Y., Su Y., Shen Q., Wang D. (2017). Langmuir.

[cit49] Zhang Q., Remsen E. E., Wooley K. L. (2000). J. Am. Chem. Soc..

[cit50] Zhang Q., Clark C. G., Wang M., Remsen E. E., Wooley K. L. (2002). Nano Lett..

[cit51] Du Z. X., Xu J. T., Fan Z. Q. (2007). Macromolecules.

[cit52] Du Z. X., Xu J. T., Fan Z. Q. (2008). Macromol. Rapid Commun..

[cit53] Su M., Huang H., Ma X., Wang Q., Su Z. (2013). Macromol. Rapid Commun..

[cit54] Wang J., Zhu W., Peng B., Chen Y. (2013). Polymer.

[cit55] Rizis G., Van De Ven T. G. M., Eisenberg A. (2014). Angew. Chem., Int. Ed..

[cit56] Rizis G., Van De Ven T. G. M., Eisenberg A. (2014). Soft Matter.

[cit57] Wu J., Weng L.-T., Qin W., Liang G., Zhong Tang B. (2015). ACS Macro Lett..

[cit58] Xu Z., Lu C., Lindenberger C., Cao Y., Wulff J. E., Moffitt M. G. (2017). ACS Omega.

[cit59] Ganda S., Dulle M., Drechsler M., Förster B., Förster S., Stenzel M. H. (2017). Macromolecules.

[cit60] Wang J., Lu Y., Chen Y. (2019). Polymer.

[cit61] Zhang J., Wang L. Q., Wang H., Tu K. (2006). Biomacromolecules.

[cit62] Finnegan J. R., He X., Street S. T. G., Garcia-Hernandez J. D., Hayward D. W., Harniman R. L., Richardson R. M., Whittell G. R., Manners I. (2018). J. Am. Chem. Soc..

[cit63] Mihut A. M., Chiche A., Drechsler M., Schmalz H., Di Cola E., Krausch G., Ballauff M. (2009). Soft Matter.

[cit64] Mihut A. M., Drechsler M., Möller M., Ballauff M. (2010). Macromol. Rapid Commun..

[cit65] Xu J. T., Fairclough J. P. A., Mai S. M., Ryan A. J. (2003). J. Mater. Chem..

[cit66] Wang M. J., Wang H., Chen S. C., Chen C., Liu Y. (2015). Langmuir.

[cit67] Wang H., Liu C. L., Wu G., Chen S. C., Song F., Wang Y. Z. (2013). Soft Matter.

[cit68] Huang W., Wang M. J., Liu C. L., You J., Chen S. C., Wang Y. Z., Liu Y. (2014). J. Mater. Chem. A.

[cit69] Schmalz H., Schmelz J., Drechsler M., Yuan J., Walther A., Schweimer K., Mihut A. M. (2008). Macromolecules.

[cit70] Yin L., Hillmyer M. A. (2011). Macromolecules.

[cit71] Schmelz J., Karg M., Hellweg T., Schmalz H. (2011). ACS Nano.

[cit72] Yin L., Lodge T. P., Hillmyer M. A. (2012). Macromolecules.

[cit73] Li Z., Liu R., Mai B., Wang W., Wu Q., Liang G., Gao H., Zhu F. (2013). Polymer.

[cit74] Wang H., Wu C., Xia G., Ma Z., Mo G., Song R. (2015). Soft Matter.

[cit75] Fan B., Liu L., Li J. H., Ke X. X., Xu J. T., Du B. Y., Fan Z. Q. (2016). Soft Matter.

[cit76] Schöbel J., Karg M., Rosenbach D., Krauss G., Greiner A., Schmalz H. (2016). Macromolecules.

[cit77] Fan B., Wang R. Y., Wang X. Y., Xu J. T., Du B. Y., Fan Z. Q. (2017). Macromolecules.

[cit78] He Q., Yuan Y., Chen F., Ma Z., Zhu X., Song R. (2017). Polymer.

[cit79] Xu S., Zhang C., Li L., Zheng S. (2017). Polymer.

[cit80] Fan B., Xue J.-Q., Guo X.-S., Cao X.-H., Wang R.-Y., Xu J.-T., Du B.-Y., Fan Z.-Q. (2018). Macromolecules.

[cit81] Ding L., Qian J., Zhu G., Li W., Zhao C., Xu Y., Mu J. (2019). J. Polym. Res..

[cit82] Qian J., Ding L., Zhu G., Wu X., Li W., Zhao C., Mu J. (2019). J. Polym. Res..

[cit83] Zhu G., Cheng S., Hu Y., Li W., Mu J. (2020). J. Polym. Res..

[cit84] Radulescu A., Mathers R. T., Coates G. W., Richter D., Fetters L. J. (2004). Macromolecules.

[cit85] Sevgen E., Dolejsi M., Nealey P. F., Hubbell J. A., De Pablo J. J. (2018). Macromolecules.

[cit86] Brubaker C. E., Velluto D., Demurtas D., Phelps E. A., Hubbell J. A. (2015). ACS Nano.

[cit87] Chen J., Yu C., Shi Z., Yu S., Lu Z., Jiang W., Zhang M., He W., Zhou Y., Yan D. (2015). Angew. Chem., Int. Ed..

[cit88] Lee C. U., Smart T. P., Guo L., Epps T. H., Zhang D. (2011). Macromolecules.

[cit89] Wei Y., Tian J., Zhang Z., Zhu C., Sun J., Li Z. (2019). Macromolecules.

[cit90] Sun J., Jiang X., Lund R., Downing K. H., Balsara N. P., Zuckermann R. N. (2016). Proc. Natl. Acad. Sci. U. S. A..

[cit91] Jiang N., Yu T., Darvish O. A., Qian S., Mkam Tsengam I. K., John V., Zhang D. (2019). Macromolecules.

[cit92] Shi Z., Wei Y., Zhu C., Sun J., Li Z. (2018). Macromolecules.

[cit93] Wang Z., Lin M., Bonduelle C., Li R., Shi Z., Zhu C., Lecommandoux S., Li Z., Sun J. (2020). Biomacromolecules.

[cit94] Lazzari M., Scalarone D., Vazquez-Vazquez C., López-Quintela M. A. (2008). Macromol. Rapid Commun..

[cit95] Legros C., De Pauw-Gillet M. C., Tam K. C., Taton D., Lecommandoux S. (2015). Soft Matter.

[cit96] Nishimura T., Sumi N., Mukai S. A., Sasaki Y., Akiyoshi K. (2019). J. Mater. Chem. B.

[cit97] Nabiyan A., Biehl P., Schacher F. H. (2020). Macromolecules.

[cit98] Cortes M. D. L. A., De La Campa R., Valenzuela M. L., Díaz C., Carriedo G. A., Soto A. P. (2019). Molecules.

[cit99] Suárez-Suárez S., Carriedo G. A., Presa Soto A. (2016). Chem.–Eur. J..

[cit100] Wu Y., Chen L., Sun X., Xu J., Gu G., Qian J. (2017). J. Saudi Chem. Soc..

[cit101] Folgado E., Mayor M., Ladmiral V., Semsarilar M. (2020). Molecules.

[cit102] Shen L., Guo H., Zheng J., Wang X., Yang Y., An Z. (2018). ACS Macro Lett..

[cit103] Li X., Jin B., Gao Y., Hayward D. W., Winnik M. A., Luo Y., Manners I. (2016). Angew. Chem., Int. Ed..

[cit104] Li X., Gao Y., Xing X., Liu G. (2013). Macromolecules.

[cit105] Gao Y., Li X., Hong L., Liu G. (2012). Macromolecules.

[cit106] Guan S., Zhang C., Wen W., Qu T., Zheng X., Zhao Y., Chen A. (2018). ACS Macro Lett..

[cit107] Guan S., Wen W., Yang Z., Chen A. (2020). Macromolecules.

[cit108] Jia L., Lévy D., Durand D., Impéror-Clerc M., Cao A., Li M. H. (2011). Soft Matter.

[cit109] Gao L., Gao H., Lin J., Wang L., Wang X. S., Yang C., Lin S. (2020). Macromolecules.

[cit110] Lin Y., Thomas M. R., Gelmi A., Leonardo V., Pashuck E. T., Maynard S. A., Wang Y., Stevens M. M. (2017). J. Am. Chem. Soc..

[cit111] Kamps A. C., Fryd M., Park S. J. (2012). ACS Nano.

[cit112] Hayward D. W., Lunn D. J., Seddon A., Finnegan J. R., Gould O. E. C., Magdysyuk O., Manners I., Whittell G. R., Richardson R. M. (2018). Macromolecules.

[cit113] Agbolaghi S., Zenoozi S., Abbasi F. (2018). J. Iran. Chem. Soc..

[cit114] Park S. J., Kang S. G., Fryd M., Saven J. G., Park S. J. (2010). J. Am. Chem. Soc..

[cit115] Lee E., Hammer B., Kim J. K., Page Z., Emrick T., Hayward R. C. (2011). J. Am. Chem. Soc..

[cit116] Kynaston E. L., Gould O. E. C., Gwyther J., Whittell G. R., Winnik M. A., Manners I. (2015). Macromol. Chem. Phys..

[cit117] Lin C. H., Tung Y. C., Ruokolainen J., Mezzenga R., Chen W. C. (2008). Macromolecules.

[cit118] Tian Y., Chen C. Y., Yip H. L., Wu W. C., Chen W. C., Jen A. K. Y. (2010). Macromolecules.

[cit119] Tung Y. C., Wu W. C., Chen W. C. (2006). Macromol. Rapid Commun..

[cit120] Yoon K. Y., Lee I. H., Choi T. L. (2014). RSC Adv..

[cit121] Yoon K. Y., Lee I. H., Kim K. O., Jang J., Lee E., Choi T. L. (2012). J. Am. Chem. Soc..

[cit122] Ryu J. H., Lee M. (2005). J. Am. Chem. Soc..

[cit123] Li K., Wang Q. (2005). Chem. Commun..

[cit124] Ledere P., Calderone A., Marsitzky D., Francke V., Geerts Y., Müllen K., Brédas J. L., Lazzaroni R. (2000). Adv. Mater..

[cit125] Urban V., Wang H. H., Thiyagarajan P., Littrell K. C., Wang H. B., Yu L. (2000). J. Appl. Crystallogr..

[cit126] Feng C., Jose Gonzalez-Alvarez M., Song Y., Li I., Zhao G., Molev G., Guerin G., Walker G., Scholes G. D., Manners I., Winnik M. A. (2014). Soft Matter.

[cit127] Wang H., Hau Wang H., Urban V. S., Littrell K. C., Thiyagarajan P., Yu L. (2000). J. Am. Chem. Soc..

[cit128] Wang H., You W., Jiang P., Yu L., Wang H. H. (2004). Chem.–Eur. J..

[cit129] Schick C., Androsch R., Schmelzer J. W. P. (2017). J. Phys.: Condens. Matter.

[cit130] Sun L., Pitto-Barry A., Kirby N., Schiller T. L., Sanchez A. M., Dyson M. A., Sloan J., Wilson N. R., O'Reilly R. K., Dove A. P. (2014). Nat. Commun..

[cit131] Presa-Soto D., Carriedo G. A., de la Campa R., Presa Soto A. (2016). Angew. Chem., Int. Ed..

[cit132] Hsiao M. S., Yusoff S. F. M., Winnik M. A., Manners I. (2014). Macromolecules.

[cit133] Shen L., Wang H., Guerin G., Wu C., Manners I., Winnik M. A. (2008). Macromolecules.

[cit134] Lee I.-H., Amaladass P., Yoon K.-Y., Shin S., Kim Y.-J., Kim I., Lee E., Choi T.-L. (2013). J. Am. Chem. Soc..

[cit135] Hurst P. J., Rakowski A. M., Patterson J. P. (2020). Nat. Commun..

[cit136] Oliver A. M., Gwyther J., Boott C. E., Davis S., Pearce S., Manners I. (2018). J. Am. Chem. Soc..

[cit137] Yin R., Sahoo D., Xu F., Huang W., Zhou Y. (2020). Polym. Chem..

[cit138] Lee I.-H., Amaladass P., Choi I., Bergmann V. W., Weber S. A. L., Choi T.-L. (2016). Polym. Chem..

[cit139] Boott C. E., Gwyther J., Harniman R. L., Hayward D. W., Manners I. (2017). Nat. Chem..

[cit140] Wang X., Guerin G., Wang H., Wang Y., Manners I., Winnik M. A. (2007). Science.

[cit141] Gilroy J. B., Gädt T., Whittell G. R., Chabanne L., Mitchels J. M., Richardson R. M., Winnik M. A., Manners I. (2010). Nat. Chem..

[cit142] Hudson Z. M., Boott C. E., Robinson M. E., Rupar P. A., Winnik M. A., Manners I. (2014). Nat. Chem..

[cit143] Yu B., Jiang X., Yin J. (2014). Macromolecules.

[cit144] Qiu H., Gao Y., Boott C. E., Gould O. E. C., Harniman R. L., Miles M. J., Webb S. E. D., Winnik M. A., Manners I. (2016). Science.

[cit145] He X., Hsiao M. S., Boott C. E., Harniman R. L., Nazemi A., Li X., Winnik M. A., Manners I. (2017). Nat. Mater..

[cit146] Wang H., Lin W., Fritz K. P., Scholes G. D., Winnik M. A., Manners I. (2007). J. Am. Chem. Soc..

[cit147] Qiu H., Russo G., Rupar P. A., Chabanne L., Winnik M. A., Manners I. (2012). Angew. Chem., Int. Ed..

[cit148] Qiu H., Hudson Z. M., Winnik M. A., Manners I. (2015). Science.

[cit149] Rupar P. A., Chabanne L., Winnik M. A., Manners I. (2012). Science.

[cit150] Hudson Z. M., Lunn D. J., Winnik M. A., Manners I. (2014). Nat. Commun..

[cit151] Finnegan J. R., Lunn D. J., Gould O. E. C., Hudson Z. M., Whittell G. R., Winnik M. A., Manners I. (2014). J. Am. Chem. Soc..

[cit152] Lunn D. J., Finnegan J. R., Manners I. (2015). Chem. Sci..

[cit153] Xu J., Zhou H., Yu Q., Manners I., Winnik M. A. (2018). J. Am. Chem. Soc..

[cit154] Nazemi A., He X., Macfarlane L. R., Harniman R. L., Hsiao M. S., Winnik M. A., Faul C. F. J., Manners I. (2017). J. Am. Chem. Soc..

[cit155] Rupar P. A., Cambridge G., Winnik M. A., Manners I. (2011). J. Am. Chem. Soc..

[cit156] Qiu H., Cambridge G., Winnik M. A., Manners I. (2013). J. Am. Chem. Soc..

[cit157] Jia L., Petretic A., Molev G., Guerin G., Manners I., Winnik M. A. (2015). ACS Nano.

[cit158] Jia L., Zhao G., Shi W., Coombs N., Gourevich I., Walker G. C., Guerin G., Manners I., Winnik M. A. (2014). Nat. Commun..

[cit159] Qiu H., Gao Y., Du V. A., Harniman R., Winnik M. A., Manners I. (2015). J. Am. Chem. Soc..

[cit160] Gao Y., Qiu H., Zhou H., Li X., Harniman R., Winnik M. A., Manners I. (2015). J. Am. Chem. Soc..

[cit161] Qiu H., Du V. A., Winnik M. A., Manners I. (2013). J. Am. Chem. Soc..

[cit162] Li X., Gao Y., Boott C. E., Winnik M. A., Manners I. (2015). Nat. Commun..

[cit163] Li X., Gao Y., Harniman R., Winnik M., Manners I. (2016). J. Am. Chem. Soc..

[cit164] Li X., Gao Y., Boott C. E., Hayward D. W., Harniman R., Whittell G. R., Richardson R. M., Winnik M. A., Manners I. (2016). J. Am. Chem. Soc..

[cit165] Petzetakis N., Dove A. P., O'Reilly R. K. (2011). Chem. Sci..

[cit166] Sun L., Petzetakis N., Pitto-Barry A., Schiller T. L., Kirby N., Keddie D. J., Boyd B. J., O'Reilly R. K., Dove A. P. (2013). Macromolecules.

[cit167] He Y., Eloi J.-C., Harniman R. L., Richardson R. M., Whittell G. R., Mathers R. T., Dove A. P., O'Reilly R. K., Manners I. (2019). J. Am. Chem. Soc..

[cit168] Schmelz J., Schedl A. E., Steinlein C., Manners I., Schmalz H. (2012). J. Am. Chem. Soc..

[cit169] Arno M. C., Inam M., Coe Z., Cambridge G., Macdougall L. J., Keogh R., Dove A. P., O'Reilly R. K. (2017). J. Am. Chem. Soc..

[cit170] He W. N., Zhou B., Xu J. T., Du B. Y., Fan Z. Q. (2012). Macromolecules.

[cit171] He X., Finnegan J. R., Hayward D. W., Macfarlane L. R., Harniman R. L., Manners I. (2020). Macromolecules.

[cit172] Xu L., Wang C., Li Y. X., Xu X. H., Zhou L., Liu N., Wu Z. Q. (2020). Angew. Chem., Int. Ed..

[cit173] Fukui T., Garcia-Hernandez J. D., MacFarlane L. R., Lei S., Whittell G. R., Manners I. (2020). J. Am. Chem. Soc..

[cit174] Patra S. K., Ahmed R., Whittell G. R., Lunn D. J., Dunphy E. L., Winnik M. A., Manners I. (2011). J. Am. Chem. Soc..

[cit175] Gwyther J., Gilroy J. B., Rupar P. A., Lunn D. J., Kynaston E., Patra S. K., Whittell G. R., Winnik M. A., Manners I. (2013). Chem.–Eur. J..

[cit176] Kynaston E. L., Nazemi A., MacFarlane L. R., Whittell G. R., Faul C. F. J., Manners I. (2018). Macromolecules.

[cit177] Jin X. H., Price M. B., Finnegan J. R., Boott C. E., Richter J. M., Rao A., Matthew Menke S., Friend R. H., Whittell G. R., Manners I. (2018). Science.

[cit178] Tao D., Wang Z., Huang X., Tian M., Lu G., Manners I., Winnik M. A., Feng C. (2020). Angew. Chem., Int. Ed..

[cit179] Tao D., Feng C., Cui Y., Yang X., Manners I., Winnik M. A., Huang X. (2017). J. Am. Chem. Soc..

[cit180] Yang S., Choi T. L. (2020). Chem. Sci..

[cit181] Nie J., Wang Z., Huang X., Lu G., Feng C. (2020). Macromolecules.

[cit182] Bousmail D., Chidchob P., Sleiman H. F. (2018). J. Am. Chem. Soc..

[cit183] Merg A. D., Van Genderen E., Bazrafshan A., Su H., Zuo X., Touponse G., Blum T. B., Salaita K., Abrahams J. P., Conticello V. P. (2019). J. Am. Chem. Soc..

[cit184] Shin S., Menk F., Kim Y., Lim J., Char K., Zentel R., Choi T.-L. (2018). J. Am. Chem. Soc..

[cit185] Boott C. E., Leitao E. M., Hayward D. W., Laine R. F., Mahou P., Guerin G., Winnik M. A., Richardson R. M., Kaminski C. F., Whittell G. R., Manners I. (2018). ACS Nano.

[cit186] Hicks G. E. J., Jarrett-Wilkins C. N., Panchuk J. R., Manion J. G., Seferos D. S. (2020). Chem. Sci..

[cit187] Blundell D. J., Keller A., Kovacs A. J. (1966). J. Polym. Sci., Part B: Polym. Lett..

[cit188] Blundell D. J., Keller A. (1968). J. Macromol. Sci., Part B: Phys..

[cit189] Guerin G., Rupar P. A., Manners I., Winnik M. A. (2018). Nat. Commun..

[cit190] Xu J., Ma Y., Hu W., Rehahn M., Reiter G. (2009). Nat. Mater..

[cit191] Qian J., Lu Y., Chia A., Zhang M., Rupar P. A., Gunari N., Walker G. C., Cambridge G., He F., Guerin G., Manners I., Winnik M. A. (2013). ACS Nano.

[cit192] Qian J., Guerin G., Lu Y., Cambridge G., Manners I., Winnik M. A. (2011). Angew. Chem., Int. Ed..

[cit193] Chen W. Y., Li C. Y., Zheng J. X., Huang P., Zhu L., Ge Q., Quirk R. P., Lotz B., Deng L., Wu C., Thomas E. L., Cheng S. Z. D. (2004). Macromolecules.

[cit194] Li B., Li C. Y. (2007). J. Am. Chem. Soc..

[cit195] Mei S., Li C. Y. (2018). Angew. Chem..

[cit196] Gilroy J. B., Lunn D. J., Patra S. K., Whittell G. R., Winnik M. A., Manners I. (2012). Macromolecules.

[cit197] Qian J., Li X., Lunn D. J., Gwyther J., Hudson Z. M., Kynaston E., Rupar P. A., Winnik M. A., Manners I. (2014). J. Am. Chem. Soc..

[cit198] Li X., Wolanin P. J., MacFarlane L. R., Harniman R. L., Qian J., Gould O. E. C. C., Dane T. G., Rudin J., Cryan M. J., Schmaltz T., Frauenrath H., Winnik M. A., Faul C. F. J. J., Manners I. (2017). Nat. Commun..

[cit199] Jin B., Sano K., Aya S., Ishida Y., Gianneschi N., Luo Y., Li X. (2019). Nat. Commun..

[cit200] Hsiao M. S., Chen W. Y., Zheng J. X., Van Horn R. M., Quirk R. P., Ivanov D. A., Thomas E. L., Lotz B., Cheng S. Z. D. (2008). Macromolecules.

[cit201] Yu W., Foster J. C., Dove A. P., O'Reilly R. K. (2020). Macromolecules.

[cit202] Cui Y., Wang Z., Huang X., Lu G., Manners I., Winnik M. A., Feng C. (2020). Macromolecules.

[cit203] Inam M., Jones J. R., Pérez-Madrigal M. M., Arno M. C., Dove A. P., O'Reilly R. K. (2018). ACS Cent. Sci..

[cit204] Guerin G., Molev G., Rupar P. A., Manners I., Winnik M. A. (2020). Macromolecules.

[cit205] Guerin G., Molev G., Pichugin D., Rupar P. A., Qi F., Cruz M., Manners I., Winnik M. A. (2019). Macromolecules.

[cit206] Xu J., Zhou H., Yu Q., Guerin G., Manners I., Winnik M. A. (2019). Chem. Sci..

[cit207] Yang L., Tan X., Wang Z., Zhang X. (2015). Chem. Rev..

[cit208] De Greef T. F. A., Smulders M. M. J., Wolffs M., Schenning A. P. H. J., Sijbesma R. P., Meijer E. W. (2009). Chem. Rev..

[cit209] Adelizzi B., Aloi A., Markvoort A. J., Ten Eikelder H. M. M., Voets I. K., Palmans A. R. A., Meijer E. W. (2018). J. Am. Chem. Soc..

[cit210] Haedler A. T., Meskers S. C. J., Zha R. H., Kivala M., Schmidt H. W., Meijer E. W. (2016). J. Am. Chem. Soc..

[cit211] Korevaar P. A., George S. J., Markvoort A. J., Smulders M. M. J., Hilbers P. A. J., Schenning A. P. H. J., De Greef T. F. A., Meijer E. W. (2012). Nature.

[cit212] Matern J., Dorca Y., Sánchez L., Fernández G. (2019). Angew. Chem., Int. Ed..

[cit213] Besenius P. (2017). J. Polym. Sci., Part A: Polym. Chem..

[cit214] Hartlieb M., Mansfield E. D. H., Perrier S. (2020). Polym. Chem..

[cit215] Ghosh G., Dey P., Ghosh S. (2020). Chem. Commun..

[cit216] Mukhopadhyay R. D., Ajayaghosh A. (2015). Science.

[cit217] Aida T., Meijer E. W. (2020). Isr. J. Chem..

[cit218] Wehner M., Würthner F. (2020). Nat. Rev. Chem..

[cit219] Zhang W., Jin W., Fukushima T., Saeki A., Seki S., Aida T. (2011). Science.

[cit220] Ogi S., Sugiyasu K., Manna S., Samitsu S., Takeuchi M. (2014). Nat. Chem..

[cit221] Wagner W., Wehner M., Stepanenko V., Würthner F. (2019). J. Am. Chem. Soc..

[cit222] Sarkar A., Sasmal R., Empereur-Mot C., Bochicchio D., Kompella S. V. K., Sharma K., Dhiman S., Sundaram B., Agasti S. S., Pavan G. M., George S. J. (2020). J. Am. Chem. Soc..

[cit223] Fukui T., Kawai S., Fujinuma S., Matsushita Y., Yasuda T., Sakurai T., Seki S., Takeuchi M., Sugiyasu K. (2017). Nat. Chem..

[cit224] Fukui T., Uchihashi T., Sasaki N., Watanabe H., Takeuchi M., Sugiyasu K. (2018). Angew. Chem..

[cit225] Aliprandi A., Mauro M., De Cola L. (2016). Nat. Chem..

[cit226] Robinson M. E., Nazemi A., Lunn D. J., Hayward D. W., Boott C. E., Hsiao M. S., Harniman R. L., Davis S. A., Whittell G. R., Richardson R. M., De Cola L., Manners I. (2017). ACS Nano.

[cit227] Ghosh G., Ghosh T., Fernández G. (2020). ChemPlusChem.

[cit228] Robinson M. E., Lunn D. J., Nazemi A., Whittell G. R., De Cola L., Manners I. (2015). Chem. Commun..

[cit229] Wan Q., To W. P., Chang X., Che C. M. (2020). Chem.

[cit230] Ogi S., Stepanenko V., Sugiyasu K., Takeuchi M., Würthner F. (2015). J. Am. Chem. Soc..

[cit231] Greciano E. E., Sánchez L. (2016). Chem.–Eur. J..

[cit232] Pal A., Malakoutikhah M., Leonetti G., Tezcan M., Colomb-Delsuc M., Nguyen V. D., Van Der Gucht J., Otto S. (2015). Angew. Chem., Int. Ed..

[cit233] Jarrett-Wilkins C., He X., Symons H. E., Harniman R. L., Faul C. F. J., Manners I. (2018). Chem.–Eur. J..

[cit234] Zhang K., Yeung M. C. L., Leung S. Y. L., Yam V. W. W. (2017). Proc. Natl. Acad. Sci. U. S. A..

[cit235] Sarkar A., Behera T., Sasmal R., Capelli R., Empereur-Mot C., Mahato J., Agasti S. S., Pavan G. M., Chowdhury A., George S. J. (2020). J. Am. Chem. Soc..

[cit236] Kang J., Miyajima D., Mori T., Inoue Y., Itoh Y., Aida T. (2015). Science.

[cit237] Zhang W., Jin W., Fukushima T., Mori T., Aida T. (2015). J. Am. Chem. Soc..

[cit238] Ma X., Zhang Y., Zhang Y., Liu Y., Che Y., Zhao J. (2016). Angew. Chem., Int. Ed..

[cit239] Hu K., Liu Y., Xiong W., Gong Y., Che Y., Zhao J. (2019). Chem. Mater..

[cit240] Liu Y., Gong Y., Guo Y., Xiong W., Zhang Y., Zhao J., Che Y., Manners I. (2019). Chem.–Eur. J..

[cit241] Liu Y., Peng C., Xiong W., Zhang Y., Gong Y., Che Y., Zhao J. (2017). Angew. Chem., Int. Ed..

[cit242] Yu W., Liu R., Zhou Y., Gao H. (2020). ACS Cent. Sci..

[cit243] Geng Y., Dalhaimer P., Cai S., Tsai R., Tewari M., Minko T., Discher D. E. (2007). Nat. Nanotechnol..

[cit244] Toy R., Peiris P. M., Ghaghada K. B., Karathanasis E. (2014). Nanomedicine.

[cit245] Albanese A., Tang P. S., Chan W. C. W. (2012). Annu. Rev. Biomed. Eng..

[cit246] Lee H., Fonge H., Hoang B., Reilly R. M., Allen C. (2010). Mol. Pharm..

[cit247] Jiang W., Kim B. Y. S., Rutka J. T., Chan W. C. W. (2008). Nat. Nanotechnol..

[cit248] Zhao J., Lu H., Xiao P., Stenzel M. H. (2016). ACS Appl. Mater. Interfaces.

[cit249] Zhao J., Stenzel M. H. (2018). Polym. Chem..

[cit250] Nazemi A., Boott C. E., Lunn D. J., Gwyther J., Hayward D. W., Richardson R. M., Winnik M. A., Manners I. (2016). J. Am. Chem. Soc..

[cit251] Street S. T. G., He Y., Jin X. H., Hodgson L., Verkade P., Manners I. (2020). Chem. Sci..

[cit252] Arno M. C., Inam M., Weems A. C., Li Z., Binch A. L. A., Platt C. I., Richardson S. M., Hoyland J. A., Dove A. P., O'Reilly R. K. (2020). Nat. Commun..

[cit253] Yu Q., Roberts M. G., Pearce S., Oliver A. M., Zhou H., Allen C., Manners I., Winnik M. A. (2019). Macromolecules.

[cit254] Dou H., Li M., Qiao Y., Harniman R., Li X., Boott C. E., Mann S., Manners I. (2017). Nat. Commun..

[cit255] Zhu W., Peng B., Wang J., Zhang K., Liu L., Chen Y. (2014). Macromol. Biosci..

[cit256] Li Z., Zhang Y., Wu L., Yu W., Wilks T. R., Dove A. P., Ding H. M., O'Reilly R. K., Chen G., Jiang M. (2019). ACS Macro Lett..

[cit257] Inam M., Foster J. C., Gao J., Hong Y., Du J., Dove A. P., O'Reilly R. K. (2019). J. Polym. Sci., Part A: Polym. Chem..

[cit258] Li Z., Sun L., Zhang Y., Dove A. P., O'Reilly R. K., Chen G. (2016). ACS Macro Lett..

[cit259] Ma C., Wang Z., Huang X., Lu G., Manners I., Winnik M. A., Feng C. (2020). Macromolecules.

[cit260] Yu Q., Roberts M. G., Houdaihed L., Liu Y., Ho K., Walker G., Allen C., Reilly R. M., Manners I., Winnik M. A. (2021). Nanoscale.

[cit261] Wu J., Ma G. H. (2016). Small.

[cit262] Wu G., Wang L., Zhou P., Wen P., Ma C., Huang X., Huang Y. (2018). Adv. Sci..

[cit263] Kumar S. P., Prathibha D. (2013). Int. J. Pharm. Sci. Nanotechnol..

[cit264] Wang Z., Van Oers M. C. M., Rutjes F. P. J. T., Van Hest J. C. M. (2012). Angew. Chem., Int. Ed..

[cit265] Bollhorst T., Rezwan K., Maas M. (2017). Chem. Soc. Rev..

[cit266] Thompson K. L., Williams M., Armes S. P. (2014). J. Colloid Interface Sci..

[cit267] Pera-Titus M., Leclercq L., Clacens J. M., De Campo F., Nardello-Rataj V. (2015). Angew. Chem., Int. Ed..

[cit268] Li M., Harbron R. L., Weaver J. V. M., Binks B. P., Mann S. (2013). Nat. Chem..

[cit269] Saraf S., Rathi R., Kaur C. D., Saraf S. (2011). Asian J. Sci. Res..

[cit270] Schmelz J., Pirner D., Krekhova M., Ruhland T. M., Schmalz H. (2013). Soft Matter.

[cit271] Dong B., Miller D. L., Li C. Y. (2012). J. Phys. Chem. Lett..

[cit272] Schöbel J., Burgard M., Hils C., Dersch R., Dulle M., Volk K., Karg M., Greiner A., Schmalz H. (2017). Angew. Chem., Int. Ed..

[cit273] Tian J., Zhang Y., Du L., He Y., Jin X. H., Pearce S., Eloi J. C., Harniman R. L., Alibhai D., Ye R., Phillips D. L., Manners I. (2020). Nat. Chem..

[cit274] Schmelz J., Karg M., Hellweg T., Schmalz H. (2011). ACS Nano.

[cit275] Mitschang F., Schmalz H., Agarwal S., Greiner A. (2014). Angew. Chem., Int. Ed..

[cit276] Hils C., Dulle M., Sitaru G., Gekle S., Schöbel J., Frank A., Drechsler M., Greiner A., Schmalz H. (2020). Nanoscale Adv..

[cit277] MacFarlane L. R., Shaikh H., Garcia-Hernandez J. D., Vespa M., Fukui T., Manners I. (2020). Nat. Rev. Mater..

[cit278] McGrath N., Patil A. J., Watson S. M. D., Horrocks B. R., Faul C. F. J., Houlton A., Winnik M. A., Mann S., Manners I. (2013). Chem.–Eur. J..

[cit279] El-Zubir O., Kynaston E. L., Gwyther J., Nazemi A., Gould O. E. C., Whittell G. R., Horrocks B. R., Manners I., Houlton A. (2020). Chem. Sci..

[cit280] Sim M., Shin J., Shim C., Kim M., Jo S. B., Kim J. H., Cho K. (2014). J. Phys. Chem. C.

[cit281] Fukui T., Diego Garcia-Hernandez J., MacFarlane L. R., Lei S., Whittell G. R., Manners I. (2020). J. Am. Chem. Soc..

[cit282] Sneyd A. J. , Fukui T., Palecek D., Prodhan S., Wagner I., Zhang Y., Sung J., Andaji-Garmaroudi Z., MacFarlane L. R., Garcia-Hernandez J. D., Wang L., Whittell G. R., Hodgkiss J. M., Chen K., Beljonne D., Manners I., Friend R. H. and Rao A., 2020, arXiv:2009.05989, https://arxiv.org/abs/2009.05989

[cit283] Shaikh H., Jin X. H., Harniman R. L., Richardson R. M., Whittell G. R., Manners I. (2020). J. Am. Chem. Soc..

[cit284] Qi R., Zhu Y., Han L., Wang M., He F. (2020). Macromolecules.

[cit285] Yang S., Shin S., Choi I., Lee J., Choi T.-L. (2017). J. Am. Chem. Soc..

[cit286] Han L., Fan H., Zhu Y., Wang M., Pan F., Yu D., Zhao Y., He F. (2020). CCS Chem..

[cit287] He F., Gädt T., Manners I., Winnik M. A. (2011). J. Am. Chem. Soc..

[cit288] Boott C. E. , MacFarlane L. R., Hudson Z. M., Webb S. E. D. and Manners I., unpublished results

[cit289] Cai J., Li C., Kong N., Lu Y., Lin G., Wang X., Yao Y., Manners I., Qiu H. (2019). Science.

[cit290] Zoppe J. O., Ataman N. C., Mocny P., Wang J., Moraes J., Klok H. A. (2017). Chem. Rev..

[cit291] Gao L., Lin J., Zhang L., Wang L. (2019). Nano Lett..

[cit292] Gilroy J. B., Rupar P. A., Whittell G. R., Chabanne L., Terrill N. J., Winnik M. A., Manners I., Richardson R. M. (2011). J. Am. Chem. Soc..

[cit293] Yang J. X., Fan B., Li J. H., Xu J. T., Du B. Y., Fan Z. Q. (2016). Macromolecules.

[cit294] Shu R., Zha L., Eman A. A., Hu W. (2015). J. Phys. Chem. B.

[cit295] Li C. Y. (2020). Polymer.

[cit296] Hayward D. W., Gilroy J. B., Rupar P. A., Chabanne L., Pizzey C., Winnik M. A., Whittell G. R., Manners I., Richardson R. M. (2015). Macromolecules.

[cit297] Qi H., Zhou T., Mei S., Chen X., Li C. Y. (2016). ACS Macro Lett..

